# Correlative imaging integrates electrophysiology with three-dimensional murine heart reconstruction to reveal electrical coupling between cell types

**DOI:** 10.1038/s44161-025-00728-9

**Published:** 2025-10-06

**Authors:** Francesco Giardini, Camilla Olianti, Gerard A. Marchal, Fernando Campos, Valentina Romanelli, Joshua Steyer, Josef Madl, Roberto Piersanti, Giulia Arecchi, Induja Perumal Vanaja, Valentina Biasci, Eva A. Rog-Zielinska, Gabriella Nesi, Leslie M. Loew, Elisabetta Cerbai, Stephen P. Chelko, Francesco Regazzoni, Axel Loewe, Martin J. Bishop, Marco Mongillo, Peter Kohl, Tania Zaglia, Callum M. Zgierski-Johnston, Leonardo Sacconi

**Affiliations:** 1 Institute for Experimental Cardiovascular Medicine, University Heart Center and Medical Faculty, University of Freiburg, Freiburg, Germany; 2 European Laboratory for Non-Linear Spectroscopy – LENS, Sesto Fiorentino, Italy; 3 Institute of Clinical Physiology, National Research Council (IFC-CNR), Florence, Italy; 4 School of Biomedical Engineering & Imaging Sciences, King’s College London, London, UK; 5 Department of Experimental and Clinical Medicine, University of Florence, Florence, Italy; 6 Institute of Biomedical Engineering, Karlsruhe Institute of Technology (KIT), Karlsruhe, Germany; 7 MOX, Department of Mathematics, Politecnico di Milano, Milan, Italy; 8 DiSTA – Department of Theoretical and Applied Sciences, eCampus University, Novedrate, Italy; 9 Department of Cardiac, Thoracic, Vascular Sciences and Public Health, University of Padova, Padua, Italy; 10 Department of Health Sciences, Section of Pathological Anatomy, University of Florence, Florence, Italy; 11 Center for Cell Analysis and Modeling, University of Connecticut, Farmington, CT USA; 12 Department of Neurosciences, Psychology, Drug Research and Child Health, University of Florence, Florence, Italy; 13 College of Medicine, Florida State University, Tallahassee, FL USA; 14 Department of Biomedical Sciences, University of Padova, Padua, Italy; 15 Veneto Institute of Molecular Medicine, Padua, Italy

**Keywords:** Ventricular tachycardia, Ventricular tachycardia, Wide-field fluorescence microscopy, Biomedical engineering

## Abstract

Cardiac fibrosis contributes to electrical conduction disturbances, yet its specific impact on conduction remains unclear, hindering predictive insight into cardiac electrophysiology and arrhythmogenesis. Arrhythmogenic cardiomyopathy is associated with fibrotic remodeling, and it accounts for most cases of stress-related arrhythmic sudden death. Here we develop a correlative imaging approach to integrate macroscale cardiac electrophysiology with three-dimensional microscale reconstructions of the ventricles. We apply this tool to a desmoglein-2 mutant mouse model to characterize the dynamics of conduction wavefronts and relate them to the underlying structural substrate. We observed that conduction through fibrotic tissue areas shows a frequency-dependent behavior, where conduction fails at high stimulation frequencies; this promotes reentrant arrhythmias, even in regions that were electrophysiologically inconspicuous at lower stimulation rates. We found that fibrotic areas undergo electrophysiological remodeling that acts as a low-pass filter for conduction, quantitatively explained by computational models informed by structural data. Collectively, our study provides a structure–function mapping pipeline and describes a pro-arrhythmogenic mechanism in arrhythmogenic cardiomyopathy.

## Abstract (web summary)

Giardini et al. present an imaging method that combines quantitative measurements of cardiac electrophysiology with high-resolution three-dimensional structural reconstructions, enabling the detection of arrhythmogenic electrical coupling between cardiomyocytes and non-myocytes in murine hearts.

## Main

Most congenital and acquired cardiac diseases feature structural remodeling of the myocardium, including fibrosis and loss of cardiomyocytes and/or their alignment (disarray), which can contribute to impaired cardiac electromechanical function^1^. Despite the clear correlation between fibrosis and pro-arrhythmic conduction disturbances^2^, the mechanisms linking fibrosis to electrophysiological alterations are incompletely characterized. The impact of fibrotic remodeling on conduction velocity is intricately related to several factors, including structural characteristics of the conductive tissue^3,4^, its heterocellular composition^5,6^ and the remodeling of cardiomyocyte electrophysiology^7,8^. It is increasingly recognized that these mechanisms are interdependent, and that they cannot easily be studied in isolation^9^.

Current predictive models of whole-heart electrical activity are primarily based on structural information of myocardium and fibrotic tissue regions obtained by state-of-the-art medical imaging techniques^10^. The predictive capabilities of these approaches are remarkably high, especially when integrated with altered electrophysiological characteristics in the proximity of the fibrotic lesion^11^.

A critical question arises: would high-resolution imaging of fibrosis (for example, cellular organization and fibrosis distribution at a micrometric scale) be sufficient to be able to accurately predict the conduction behavior based solely on structural information? Or is the integration of additional knowledge of electrophysiological remodeling required to correctly predict cardiac electrical activity in pathologically remodeled hearts? While of inherent fundamental interest, the answer to this question also holds important implications, as it may inform the development of innovative prediction models for arrhythmia susceptibility in individuals with cardiac fibrosis.

Assessing the impact of microstructural remodeling on electrical dysfunction in whole organs presents substantial challenges. These include the difficulty in obtaining high-resolution three-dimensional (3D) data across large tissue volumes, and the scarcity of reliable experimental ground truth data to validate predicted functional behavior. To address these challenges, we developed a fully optical, correlative approach that integrates quantitative measurements of electrical function with high-resolution 3D structural reconstructions of entire mouse ventricles. We used hearts from desmoglein-2 mutant mice, which exhibit a biventricular form of arrhythmogenic cardiomyopathy, closely resembling human disease^12^. Arrhythmogenic cardiomyopathy is an inherited heart disease that involves localized replacement of cardiomyocytes with fibrotic tissue, ventricular dysfunction and arrhythmias^13–15^. Our aim was to elucidate the interrelation of locally varying fibrosis and cardiac electrical conduction.

We performed optical mapping of action potential (AP) shapes and conduction in Langendorff-perfused hearts, apically paced at different rates. Subsequently, we fixed the hearts and used an optimized optical clearing protocol to render organs optically transparent^16,17^. We used a high-throughput light-sheet mesoscope^18^ to acquire high-resolution 3D tomographic images of the entire ventricles with a micrometric voxel size. This enabled the identification and segmentation of cardiac muscle and collagen deposition as a marker of fibrosis. We quantified 3D cardiomyocyte orientation and distinguished between compact fibrosis (CF) and non-compact fibrosis (NCF). Finally, we co-registered the 3D anatomical information with the corresponding functional mapping data. A computational model of ventricular electrical activity was then developed, incorporating the high-resolution structural data of each individual heart. This computational framework was used to investigate the utility of increasing levels of detail in structural characteristics and electrophysiological alterations in reproducing experimentally observed electrical behavior, including rate-dependent conduction through fibrotic tissue regions.

## Results

### Electrophysiological characterization

**Fig. 1:**
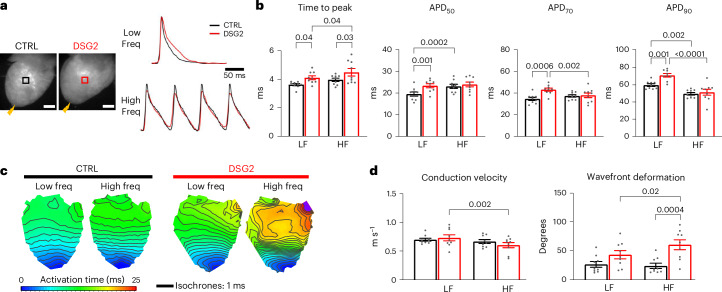
Functional characterization of AP kinetics and conduction. **a**, A CTRL and a DSG2 mouse heart during optical mapping. Scale bars, 2 mm. Hearts were electrically paced at the apex (yellow lightning bolt). Fluorescence signals (∆*F*/*F*) were extracted from the LV (black region of interest (ROI), CTRL; red ROI, DSG2) during a burst of 15 stimuli at LF (5 Hz) and HF (≥10 Hz). Freq, frequency. **b**, Average AP upstroke kinetics (TTP) and AP duration at 50%, 70% and 90% repolarization (APD_50_, APD_70_ and APD_90_, respectively) in the LV free wall of CTRL (black) and DSG2 (red) hearts. **c**, LV activation maps of a CTRL and a DSG2 heart during pacing at LF and HF, showing deformation of the advancing wavefront at HF pacing in the DSG2 heart. Activation time is color coded, and the black lines represent isochrones with 1-ms resolution. **d**, Average conduction velocity (CV, m s^−1^) and wavefront deformation (as angular dispersion, in degrees) in CTRL and DSG2 LV free walls. Data were collected from nine CTRL and nine DSG2 mouse hearts and are reported as means ± s.e.m. Two-way repeated-measures analysis of variance (ANOVA) was performed with Tukey post hoc comparison. Source data

**Extended Data Fig. 1:**
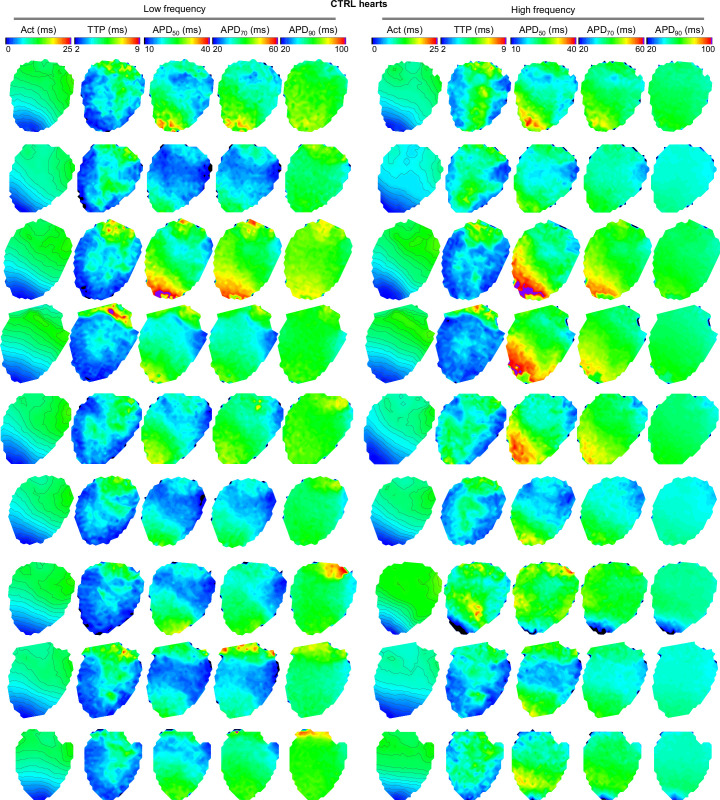
Left-ventricular maps of all included control (CTRL) hearts. Hearts were paced at LF (5 Hz; left panel) and HF ( ≥ 10 Hz; right panel). Activation time (Act) is color-coded, with isochrones of 1 ms. Time-to-peak (TTP), AP duration at 50%, 70%, and 90% of repolarization (APD50, APD70, APD90, respectively) are color-coded with the scale indicated above. Average data obtained from these maps are included in Fig. 1.

**Extended Data Fig. 2:**
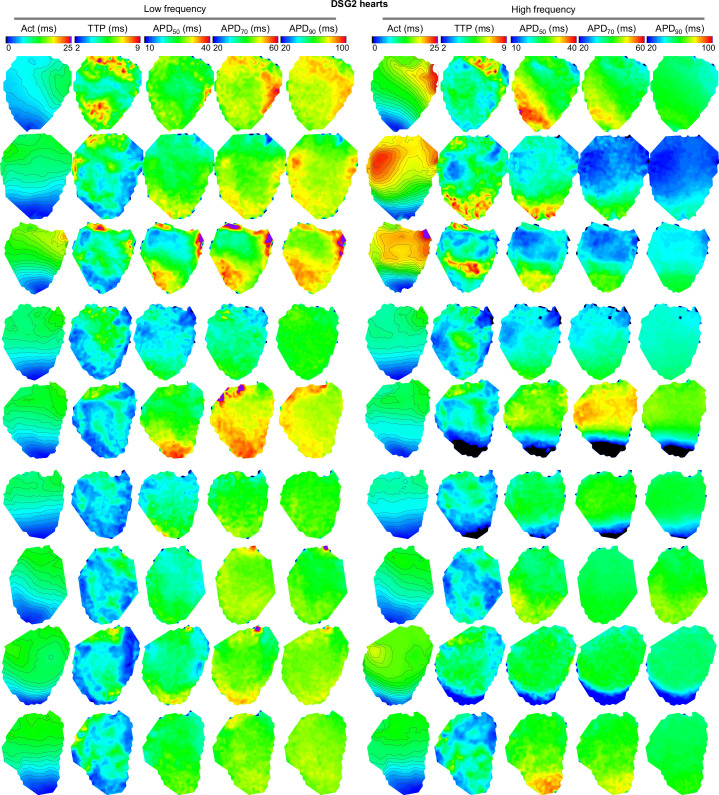
Left-ventricular maps of all included DSG2 hearts. Hearts were paced at LF (5 Hz; left panel) and HF ( ≥ 10 Hz; right panel). Activation time (Act) is color-coded, with isochrones of 1 ms. Time-to-peak (TTP), AP duration at 50%, 70%, and 90% of repolarization (APD50, APD70, APD90, respectively) are color-coded with the scale indicated above. Average data obtained from these maps are included in Fig. 1.

We optically mapped cardiomyocyte transmembrane potential to quantify AP shape and propagation kinetics across the left ventricular (LV) free walls of isolated, Langendorff-perfused hearts from *Dsg2*^mut/mut^ mice lacking functional desmoglein-2 (DSG2; *N* = 9) and littermate controls (CTRL; *N* = 9). To overdrive the sinus rhythm—4.9 Hz ± 0.2 Hz in CTRL and 5.5 Hz ± 0.3 Hz in DSG2—membrane potential was mapped while the heart was paced at the apex at a low frequency (LF) or a high frequency (HF; Fig. 1a). LF was set to 5 Hz, while HF was the fastest pacing rate that could elicit a 1:1 AP response in the individual hearts (this ranged from 10 Hz to 15 Hz). AP activation times, quantified by the time to peak (TTP), were prolonged in DSG2 hearts compared with CTRL, and longer at HF compared with LF only in DSG2. DSG2 hearts showed slower repolarization than CTRL hearts, indicated by longer AP duration at 50%, 70% and 90% repolarization (APD_50_, APD_70_ and APD_90_, respectively), when pacing at LF; no significant differences were seen between groups when pacing at HF (Fig. 1b). We investigated conduction properties in the LV free wall, assessing local conduction velocity and wavefront regularity. While conduction velocity was not significantly different between DSG2 and CTRL, DSG2 hearts exhibited slowed conduction at HF compared with LF (not seen in CTRL hearts). Wavefront deformation was assessed as the angular variability of conduction. While CTRL hearts exhibited mostly uniform propagation, with wavefronts traveling from apex to base at both stimulation frequencies, DSG2 hearts showed pronounced wavefront irregularities at HF, both in comparison to CTRL hearts and to DSG2 hearts during LF pacing (Fig. 1c,d). Thus, while LF pacing highlighted altered repolarization, HF pacing unraveled conduction defects in DSG2 hearts. Functional maps of key parameters in all samples are shown in Extended Data Figs. 1 and 2.

### Whole-ventricle reconstruction

**Fig. 2:**
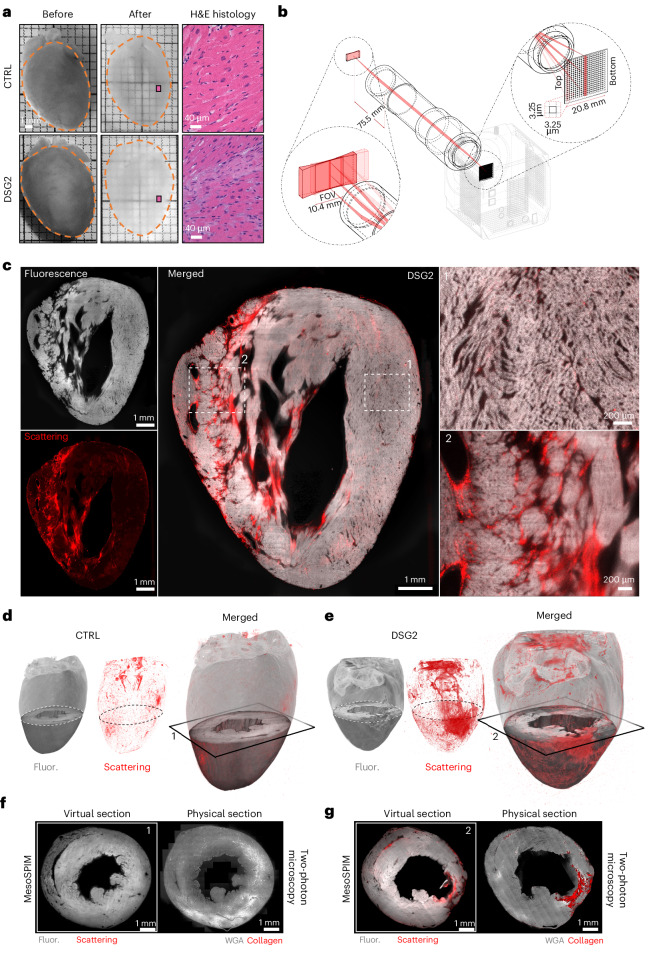
3D reconstruction of myocardium and collagen in cleared hearts. **a**, Optical clearing of entire mouse hearts by a cardiac-optimized SHIELD protocol. CTRL and DSG2 hearts are shown before (in 4% paraformaldehyde (PFA)) and after clearing (in EasyIndex solution). H&E histological inspection of cleared tissue is shown for both samples. **b**, Detection scheme of the mesoSPIM microscope, based on a custom-designed telecentric lens. Emission light from a FOV of 10.4 mm × 10.4 mm is collected by a ×2 telecentric lens and imaged on the camera sensor, working in a rolling-shutter modality that is synchronized with the position of the light-sheet focal beam as it advances through the tissue. **c**, LV optical section of a cleared DSG2 heart: myocardium fluorescence (in gray) and scattering signal (in red) were imaged with a voxel size of 3.25 × 3.25 × 3.1 µm^3^ in *XYZ*. Inset 1: the cellular organization of the myocardium. Inset 2: the extracellular matrix distribution inside a fibrotic area of the LV wall. These features were consistently observed in all analyzed samples. **d**,**e**, 3D reconstructions of entire cleared mouse ventricles (**d**, CTRL; **e**, DSG2) acquired with the mesoSPIM setup by detecting the fluorescence signal (Fluor.; gray) and scattered light (red). **f**,**g**, Correlation between mesoSPIM scattered light and collagen labeling in histological sections. Left: virtual sections of the 3D mesoSPIM reconstructions at the level indicated in **d** and **e**; right: physical sections of the same hearts from matching ventricular tissue regions, stained with wheat germ agglutinin (WGA) and for collagen (CNA35), imaged by two-photon microscopy (two CTRL and two DSG2 hearts).

After completing functional assays, all hearts were fixed and rendered fully transparent while maintaining structural preservation, using a cardiac-optimized SHIELD protocol (Fig. 2a). To assess tissue integrity at the microscopic level, histological analyses were performed on a subset (three CTRL and one DSG2) of cleared hearts. Hematoxylin and eosin (H&E) staining was applied to nonconsecutive ventricular sections, revealing the presence of transverse striations (Supplementary Fig. 1) and the characteristic alignment of cardiomyocytes, confirming the effective preservation of tissue architecture.

**Extended Data Fig. 3:**
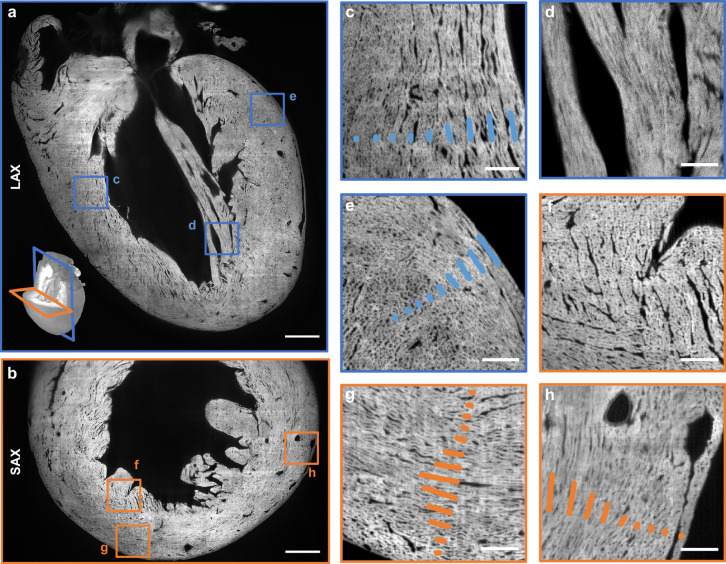
Resolving cellular orientation in the entire heart. High-resolution reconstructions by fluorescence signal of cardiac muscle of a cleared healthy heart, collected by mesoSPIM. (**a**, **b**) The long axis (LAX) plane and Short Axis (SAX) plane sections are shown. Main cellular orientation is well resolved in both LAX and SAX views, enabling detection of cellular alignment following the helical angle distribution in the myocardial wall (highlighted in **c**, **e**, **g**, and **h** panels). Cells can be resolved also in the trabeculae inside the cardiac chamber (panels **d**, **f**). Pixel size: 3.25 µm × 3.25 µm. Scale bar: 1 mm (left panels), 200 µm (C-H inserts).

The ventricles of transparent hearts were reconstructed using a mesoSPIM-based^18^ light-sheet microscope (Fig. 2b). The microscope is composed of a custom-designed telecentric lens and a high-performance back-illuminated sCMOS sensor for light collection, resulting in an object field of view (FOV) of approximately 1 cm^2^, which is sufficient to acquire an entire longitudinal cross-section of mouse hearts with negligible field-curvature distortion (Supplementary Figs. 2 and 3). Tomographic scans were generated by advancing the heart through the light sheet while detecting tissue fluorescence and elastic scattering signals, with an imaging voxel size of 3.25 × 3.25 × 3.1 µm³, later resampled to an isotropic resolution of 6 × 6 × 6 µm³ (Fig. 2c). We used the tissue fluorescence signal to create a 3D reconstruction of the myocardium in CTRL and DSG2 hearts, both at the macroscopic level (in terms of ventricular gross anatomy; Fig. 2d,e) and at the microscopic level (where myocardial morphology was detectable at the single cardiomyocyte level; Fig. 2c and Extended Data Fig. 3).

**Extended Data Fig. 4:**
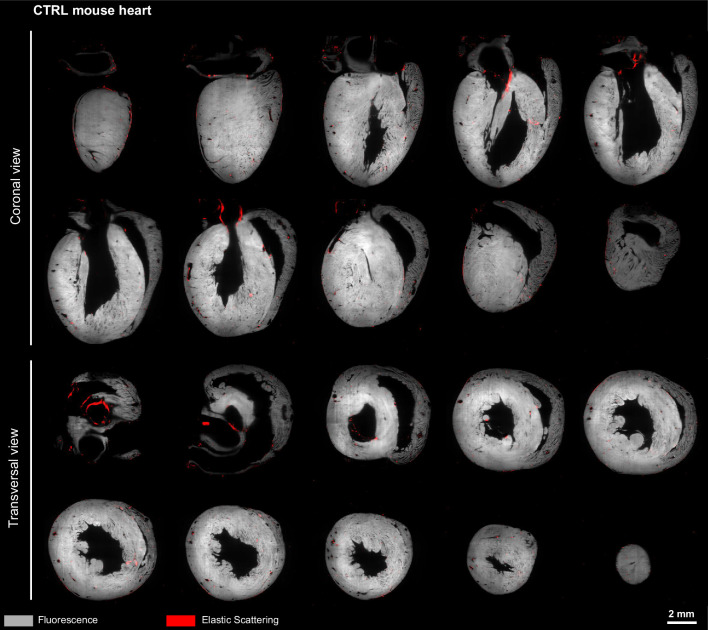
MesoSPIM tomography of a cleared mouse heart. A representative whole-heart reconstruction of a healthy mouse heart performed following the optimized SHIELD clearing protocol and the MesoSPIM-based imaging protocol. For each heart, two identical reconstructions were acquired, both with and without the long-pass filter (λ > 650 nm) to collect tissue fluorescence (here represented with a grey signal) and elastic scattering (red signal), respectively. Original images voxel size: 3.25 µm × 3.25 µm × 3.1 µm; two-channels whole-heart tomography voxel size: 6 µm × 6 µm × 6 µm.

**Extended Data Fig. 5:**
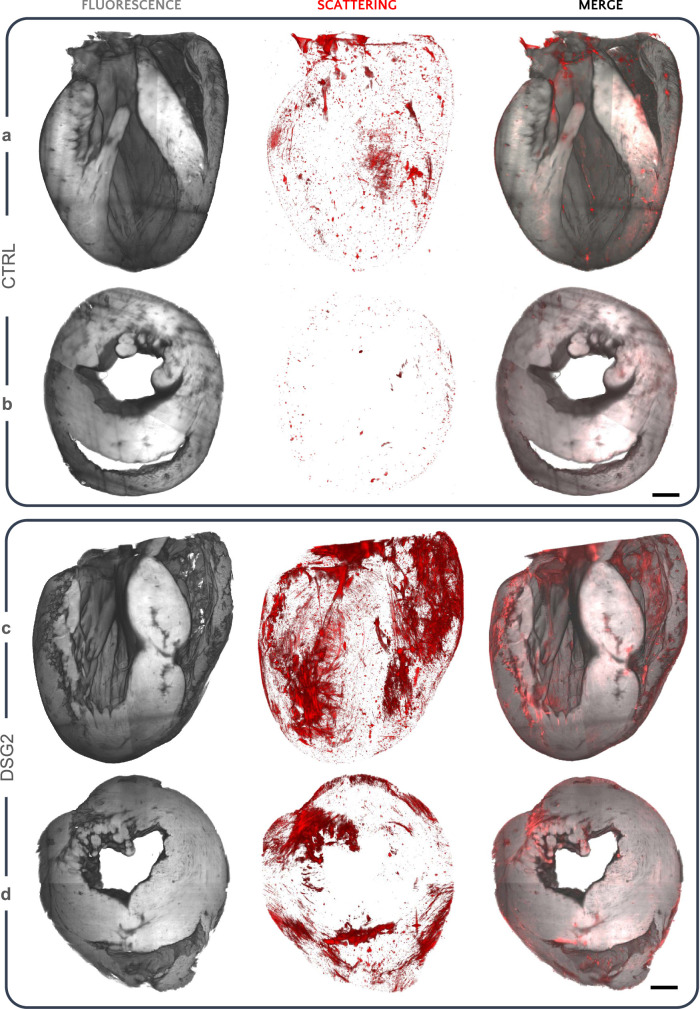
Mouse whole heart reconstruction. Coronal (**a**, **c**) and transversal (**b**, **d**) views of three-dimensional reconstructions of cleared CTRL and DSG2 entire hearts by mesoSPIM. Three-dimensional reconstructions are generated with an isotropic voxel size of 6 µm from original images (pixel size of 3.25 µm x 3.25 µm x 3.1 µm in XYZ) and rendered with ImageJ software. The final reconstructions (on the right) are generated by merging fluorescence (myocardium) and scattered light (collagen). Scale bars: 1 mm.

**Extended Data Fig. 6:**
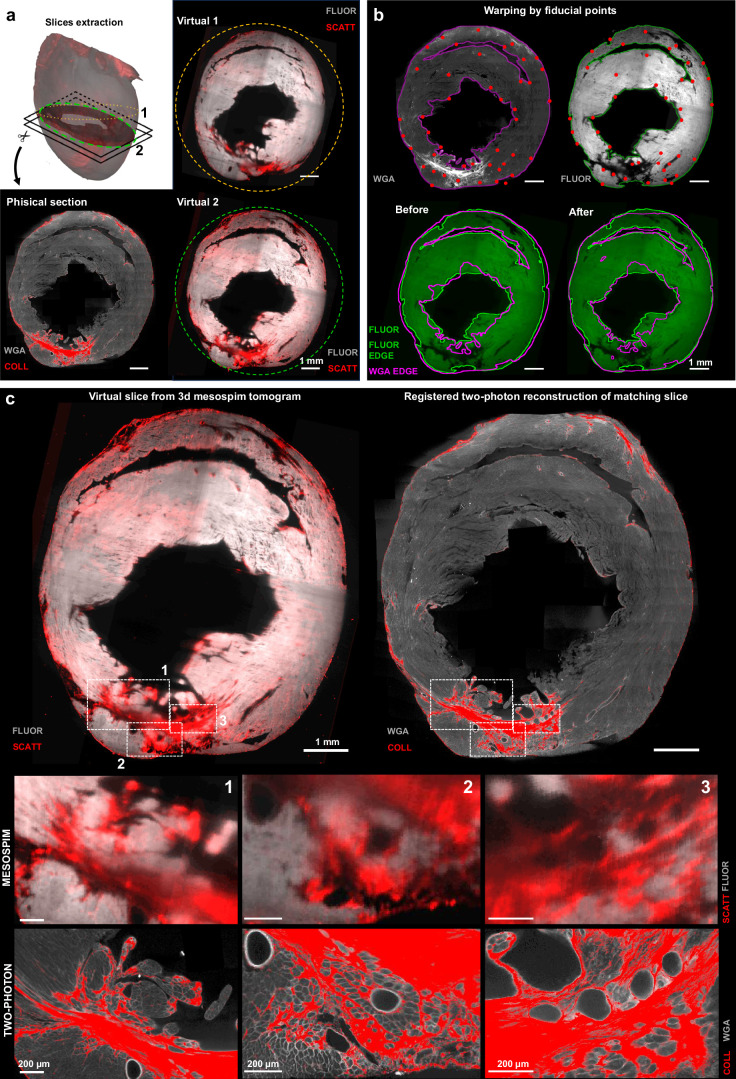
Validation of the scattering signal using two-photon microscopy. **a**, A physical transverse slice (thickness: 300 μm) excised from a cleared DSG2 heart after mesoSPIM imaging was reconstructed using two-photon microscopy. Membranes were labeled with WGA–Alexa Fluor 594 (gray), and collagen with CNA35–eGFP (red). A virtual transverse slice at the same anatomical depth (from base to apex) is shown for comparison (“Virtual 1”). A three-dimensional rotation was applied to a downsampled version of the tomogram to optimize spatial alignment between the physical and virtual slice (“Virtual 2”), correcting the inclination of the physical cut. **b**, Registration of the virtual and physical slices images was performed using elastic image warping of the two-photon data to compensate for tissue deformation due to slicing and imaging steps. Fiducial points were selected based on myocardial features (fluorescence vs. WGA signal). The borders of the segmentations from the two images are shown before and after the warping procedure. **c**, Comparison between mesoSPIM-based scattering signals and the two-photon reconstruction of the same slice after 3D alignment (panel **a**) and deformation correction (panel **b**), used as ground truth for collagen signal validation. Insets: mesoSPIM pixel size = 20 μm × 20 μm; two-photon pixel size: 1.72 μm × 1.72 μm.

In addition, we observed that the scattering signal predominantly originates from regions surrounding major vessels and valves in CTRL hearts (Extended Data Fig. 4), while in DSG2 hearts elevated scattering signals were also present in the ventricular walls, with topologies that span from diffuse signals to compact/patchy regions (Fig. 2c–e, Extended Data Fig. 5, and Supplementary Videos 1 and 2). Based on these observations and the well-known scattering strength of collagen^19,20^, we hypothesized that the scattering signal could be used to localize and quantify focally elevated extracellular matrix density. To validate this hypothesis, we co-registered and compared virtual sections from mesoSPIM tomographies with mechanically sliced sections of the same organs. Sliced sections were stained with fluorescent markers to report cell outlines and collagen distribution, imaged using two-photon microscopy, and co-registered with 3D mesoSPIM data (Fig. 2f,g and Extended Data Fig. 6). Additionally, some slices were used for analyses using Masson’s trichrome staining (Supplementary Fig. 4). These analyses confirmed the high reliability of fluorescence and scattering signals for detecting cardiac muscle and extracellular matrix, respectively, providing valuable insights into 3D tissue morphology at different scales, including the detection of compact patches of collagen and diffuse/interstitial fibrosis.

### 3D morphometry of myocardium and extracellular matrix

**Fig. 3:**
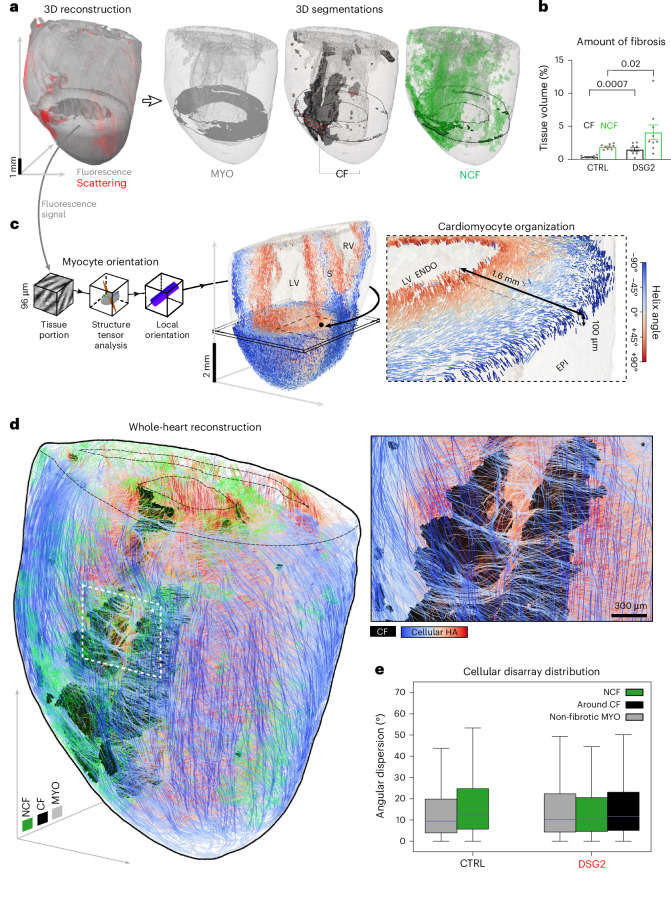
Organ-scale reconstruction of myocardium, fibrosis and 3D cardiomyocyte organization. **a**, 3D reconstruction and segmentation of a DSG2 heart: myocardium (MYO, gray) was segmented by fluorescence signal, CF (black) and NCF (green) from scattered light. A transversal plane is shown, with the outer profile of CF and NCF highlighted in red and black, respectively. **b**, Percentage of tissue classified as CF and NCF with respect to the total tissue volume in CTRL and DSG2 hearts. Data were collected from nine CTRL and nine DSG2 mouse hearts and reported as means ± s.e.m. Two-way repeated-measures ANOVA performed with Tukey post hoc comparison was applied. **c**, Schematic of the image analysis workflow developed to map the 3D cellular orientation (isotropic spatial resolution of 96 μm) from muscle fluorescence signal (isotropic voxel size of 6 μm) in whole-ventricle reconstructions. Cellular architecture of a DSG2 mouse heart is shown. Inset: a virtual slice (thickness, 100 μm), where helix angle distribution of locally prevailing cardiomyocyte orientation is color coded. LV ENDO, left ventricular endocardial surface; EPI, epicardial surface. **d**, Example of a reconstruction of a DSG2 heart where locally prevailing cardiomyocyte orientation (streamlines; color maps helix angle as reported in **c**), CF (black) and NCF (transparent green) are shown. Resolution is sufficient to detect bundles of aligned cardiomyocytes, and their rearrangement around CF in the LV free wall (inset). **e**, Box plots of the distribution of 3D cellular disarray—defined as the angular distance between the local cellular orientation and average orientation (spatial scale, 300 μm)—found in different areas of myocardium (NCF, area around CF, and non-fibrotic myocardium). Boxes show the quartiles, the median is represented by the central lines, and the whiskers extend to the farthest data point within 1.5 times the interquartile range. The area around CF is defined as tissue within 300 μm of CF. Data were collected from reconstructions of two CTRL and four DSG2 hearts. Source data

**Extended Data Fig. 7:**
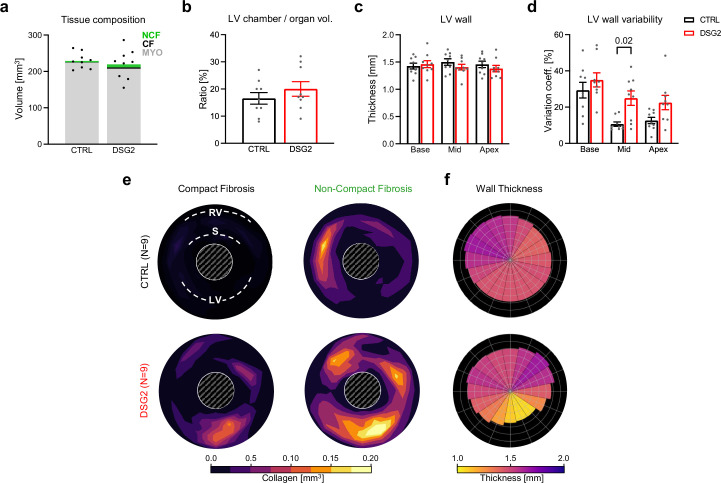
Anatomical investigation of CTRL and DSG2 hearts. **a**: Tissue composition (dots: total volume of reconstructed tissue; columns: average amount of MYO, CF, and NCF); **b**: Ratio between LV chamber lumen and myocardium volume; **c**,**d**: Mean and intra-individual variation coefficient of LV wall (free wall and interventricular septum) thickness in accordance with the AHA seventeen-segments standard protocol. **e**: Average distribution of CF and NCF in CTRL and DSG2 hearts, mapped on an ideal cardiac cross-section. Gray shaded area represents LV chamber lumen; the dashed lines represent the septum (S), LV, and right ventricle (RV) free wall positions. **f**: Wall thickness measured in the mid-cavity cross section, average between samples and reported in the same reference system of panel **e**. Data was collected from 9 CTRL and 9 DSG2 mouse hearts and reported as mean ± S.E.M. Two-way RM ANOVA analysis performed with Tukey post-hoc correction was applied. Source data

All cardiac tomograms were segmented into three distinct tissue classes, based on the fluorescence and scattering signal (Fig. 3a and Supplementary Video 3): cardiac muscle (in gray), NCF (in green, including interstitial and diffuse fibrosis) and CF (in black, defined as compact patches of extracellular matrix). Detailed 3D morphometric analyses revealed diffuse myocardial replacement by fibrotic tissue in DSG2 hearts, without evidence of hypertrophy (Extended Data Fig. 7a). LV chamber dimensions, including chamber volume (Extended Data Fig. 7b) and average wall thickness (encompassing the free wall and interventricular septum; Extended Data Fig. 7c), were comparable between DSG2 and CTRL hearts across basal, mid-cavity and apical transverse sections. However, within individual DSG2 hearts, the ventricular wall thickness was more variable in the mid-cavity section (Extended Data Fig. 7d). As expected, we found higher levels of CF and NCF in DSG2 hearts, compared with CTRL (Fig. 3b), predominantly localized in the LV free wall (Extended Data Fig. 7e), where free wall thinning was also detected (Extended Data Fig. 7f). Moreover, CF was most prominent in the mid-myocardial layers of LV free wall and septum, while NCF was more evenly distributed transmurally, usually surrounding regions of CF (Supplementary Video 3).

In addition to fibrosis, cardiac conduction can also be influenced by the organization of cardiomyocytes, since propagation preferentially follows their locally prevalent direction. This complex organization spans from the macroscale down to the microscale, including laminar sheetlets and strands of well-aligned myocytes, whose locally prevailing orientation is usually referred to as ‘cardiac fiber orientation’. To assess the myocyte arrangement, we developed an image analysis software for mapping the prevalent orientation of cells throughout the ventricles (including free walls and interventricular septum; Fig. 3c). This approach allowed us to reliably extract the local 3D orientation of cells, providing an overall reconstruction of cardiac fiber orientation across whole ventricles and reconfirming the known helix angle distribution between the epicardial and endocardial surfaces (Supplementary Videos 4 and 5). By superimposing cardiomyocyte organization with extracellular matrix in DSG2 hearts (Fig. 3d), we spatially correlated the distribution of CF and NCF with cardiomyocyte organization, emphasizing the reorganization of cardiac muscle around fibrotic patches (Supplementary Video 6).

**Extended Data Fig. 8:**
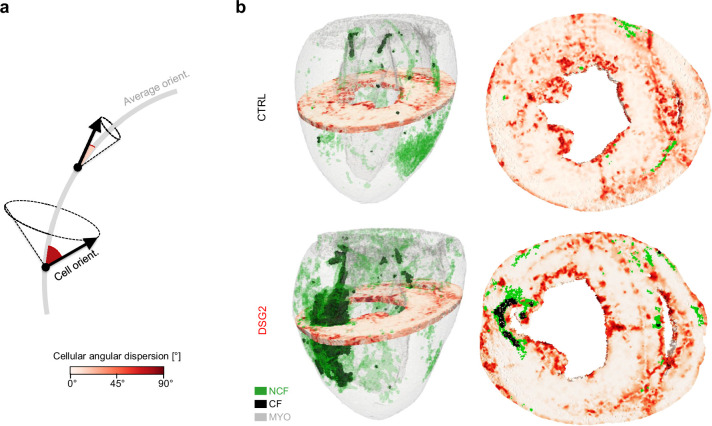
Three-dimensional mapping of cardiomyocyte disarray. **a**: Schematic workflow of 3D cellular disarray quantification defined as the angular distance between cellular orientation and average orientation (spatial scale: 300 μm). **b**: Transversal sections of cellular disarray maps in one CTRL and one DSG2 hearts.

We further quantified the misalignment of myocytes within virtual voxels measuring 300 µm per side, creating a 3D map of the cellular disarray (Extended Data Fig. 8). By applying this analysis on a subset of hearts (two CTRL hearts showing no wavefront fragmentation and four DSG2 hearts showing a pacing-rate dependency of wavefront propagation), we found that the degree of cellular disarray does not differ between DSG2 and CTRL hearts. High subendocardial and subepicardial disarray values may be related to the complex arrangement of endocardial trabeculae and to the presence of coronary vessels on the epicardial LV free wall. We did not observe a spatial correlation of cellular disarray with fibrosis, as no differences were found between areas of myocardium adjacent to CF, within NCF or in areas without detectable fibrosis (Fig. 3e).

### Morpho-functional correlation

**Fig. 4:**
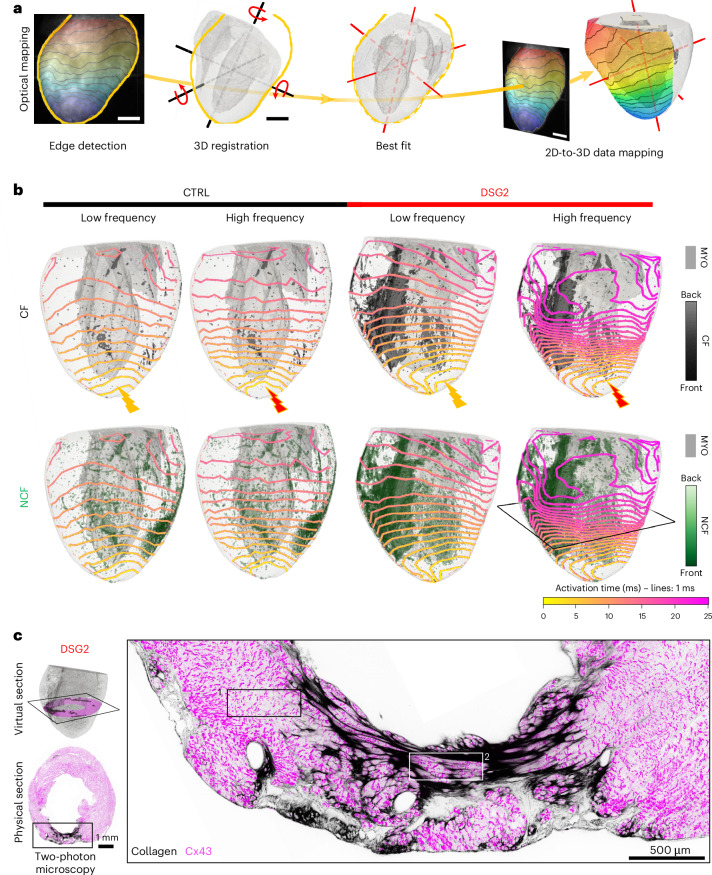
Morpho-functional correlation between conduction and fibrosis distribution. **a**, Registration pipeline between functional and structural data: the ventricular shape (in yellow) is extracted from optical mapping images. 3D mesoSPIM-based tomographs are automatically oriented to match this shape (dashed gray outline). The 2D functional data (activation maps) are then mapped onto the appropriately rotated 3D mesh. **b**, Ventricular activation maps of Fig. 1c correlated with CF (first row, in black) and with NCF (second row, in green) surrounding CF. Isochrone resolution, 1 ms. Only the fibrosis found in the frontal free wall is shown, and its depth is indicated by color intensity. **c**, Transmural fibrotic area in the transversal section of the DSG2 heart shown in **b**, reconstructed with two-photon microscopy (black, collagen; magenta, Cx43). Cx43 punctae are visible in myocardial tissue surrounding fibrosis (1) and in strands of cells inside CF (2) (observed in two DSG2 hearts).

After independently analyzing the electrophysiological and structural properties of CTRL and DSG2 hearts, we co-registered and projected two-dimensional (2D) functional data onto the corresponding 3D anatomical meshes, thus generating a 1:1 morpho-functional correlation for each heart (Fig. 4a, Supplementary Figs. 5 and 6, and Supplementary Video 7) with a spatial accuracy of ≈85 µm (Supplementary Fig. 5f) as established by a dedicated registration pipeline. We focused on correlating the spatial distribution of CF and NCF with activation maps at LF and HF pacing (Fig. 4b). CTRL hearts did not contain areas of CF, and they displayed linear conduction patterns both at LF and HF pacing. In contrast, while areas of fibrosis in DSG2 hearts were not associated with detectable irregularities in conduction patterns during LF pacing, conduction slowing was observed at HF in the areas of transition from myocardium into fibrotic areas.

Propagation through fibrotic areas during LF pacing may be explained by the non-transmural nature of CF (Extended Data Fig. 6), which may give rise to excitation in cardiomyocytes at different transmural depths (not necessarily at the level of the fibrotic layers), combined with the presence of conductive cells (cardiomyocyte and/or non-myocyte) within fibrotic patches. To further investigate the structural basis of conduction across fibrotic tissue, we reconstructed and analyzed at high resolution collagen and connexin-43 (Cx43) on transverse sections from two DSG2 hearts, revealing the presence of Cx43-expressing cells within patches of CF (Fig. 4c).

**Fig. 5:**
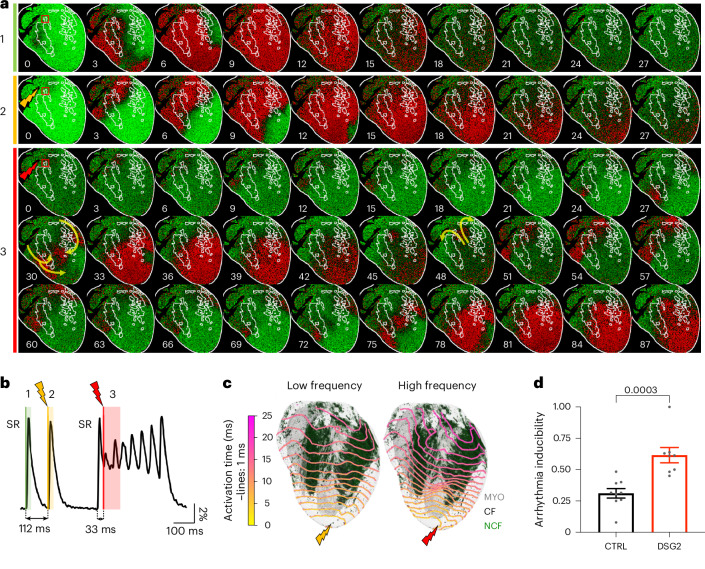
Impact of fibrosis on conduction and arrhythmogenesis during extra-beat electrical stimulation. **a**, Frames of optical mapping (Δ*F*/*F*_0_) of one sinus beat (number 1), two stimulated extra beats (number 2 and number 3, 112 ms and 33 ms after the preceding sinus beat, respectively) applied to the right ventricular outflow tract (yellow and red lightning bolts) of a DSG2 heart during an arrhythmia induction protocol. The red panel shows a reentrant arrhythmia induced by trigger number 3. Activated tissue (during AP) is indicated in red and resting tissue (excitable) in green. Structural reconstruction of CF (white profile outlines) is superimposed. **b**, Fluorescence signal (extracted from the ROI shown in **a**, red square), showing transmembrane potential dynamics during the extra-beat stimulations in **a**. Colored rectangles indicate time windows selected for representative frames in **a**. Below: black arrows indicate the time interval between last sinus beat and the extra trigger. Extra-beat number 3 gave rise to a non-sustained ventricular tachycardia that revolves around the fibrotic region. **c**, Activation maps (isochrones resolution of 1 ms) of the same DSG2 heart shown in **a** upon apical pacing at LF and HF, mapped on the 3D reconstruction (gray, myocardium, MYO; black, CF; green, NCF). Conduction through the CF tissue regions is slowed at HF pacing. **d**, Arrhythmia inducibility, provoked by extra beats triggered within 75 ms from the preceding sinus beat excitation in CTRL (black) and DSG2 (red) hearts. Data were collected from nine CTRL and eight DSG2 mouse hearts and are reported as means ± s.e.m. Mann–Whitney test (two-tailed) was applied. Source data

We found that fibrotic structures that support conduction at LF can slow or obstruct conduction at HF, suggesting that wavefront fragmentation may be evoked in conditions such as ventricular tachycardia or extrasystole. To investigate this possibility, we analyzed optical mapping experiments where extrasystoles were triggered as extra beats by electrical stimulation at the right ventricular outflow tract during sinus rhythm (Fig. 5). By aligning this data with the corresponding fibrosis reconstructions, we found that conduction through areas of fibrosis is present during the sinus rhythm (Fig. 5a) and when the extra stimulus occurs with a delay of at least 75–80 ms after the preceding sinus beat (Fig. 5a). At a shorter delay (Fig. 5a), conduction fails in the proximity of the CF, inducing a non-sustained ventricular tachycardia, in which the activation wavefront is deformed by the fibrotic tissue (Supplementary Videos 8 and 9, showing different examples of DSG2 hearts).

These observations are consistent with a pacing-rate dependence of the conduction through fibrotic tissue observed in DSG2 hearts during apical pacing (Fig. 5c). In line with this, we found that extra stimuli triggered at the right ventricular outflow tract with intervals shorter than 75 ms from the sinus beat induced ventricular tachycardia more frequently in DSG2 compared with CTRL hearts (Fig. 5d).

### Integrative computational modeling

**Fig. 6:**
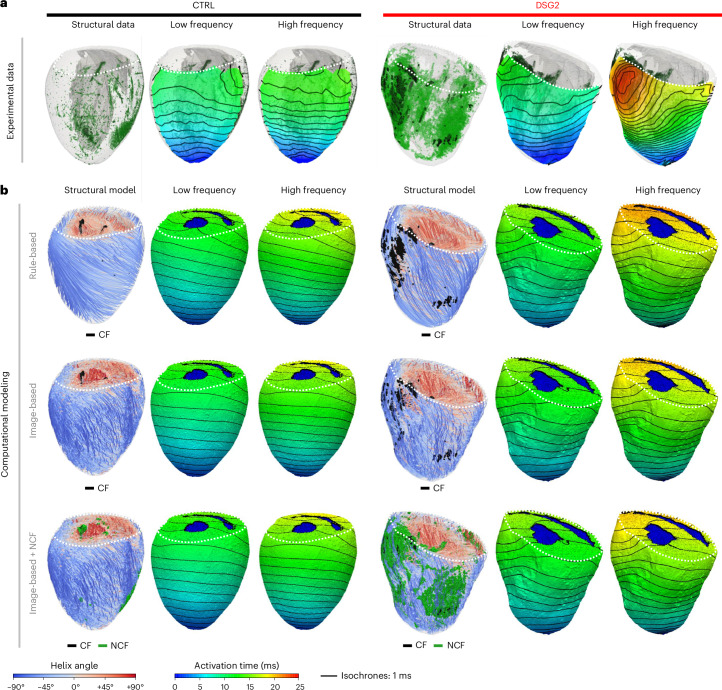
Integrated computational modeling of AP propagation. **a**, Activation maps measured via optical mapping during apical pacing at LF (5 Hz) and HF (15 Hz) in 1 CTRL and 1 DSG2 heart were projected onto the corresponding 3D structural data. **b**, Electrical propagation during apical pacing at LF and HF, simulated using a functional model of electrical activity in the same hearts as in **a**. For each sample, structural information was integrated into a biventricular geometrical model (spatial resolution, 100 μm) at 3 progressive levels: a theoretical cardiomyocyte distribution (rule-based), actual cardiomyocyte organization (image-based) and with the addition of NCF distribution. 3D activation maps of the last beat (after ten pulses at LF and HF) are shown. The ventricular cavity orifice is displayed in blue for clarity.

We used computational modeling to investigate the functional impact of structural properties, assessing whether the presence of CF patches, regions of NCF or cardiomyocyte organization at macroscales and microscales may quantitatively explain the wavefront deformations observed in DSG2 hearts at HF. We integrated monodomain representations of cardiomyocyte excitation and conduction in anatomical models of one CTRL heart and two DSG2 hearts (the latter experimentally showing pacing-rate dependency of conduction through fibrosis), incorporating levels of fibrosis and cardiomyocyte orientation maps. To facilitate direct comparison between experimental functional data (surface maps; Fig. 6a) and in silico (intrinsically 3D; Fig. 6b), the simulated myocyte membrane potentials were averaged across the entire depth of the LV free wall, aiming to approximate the experimental activation maps, where transmural fluorescence contributions are integrated^21^.

**Extended Data Fig. 9:**
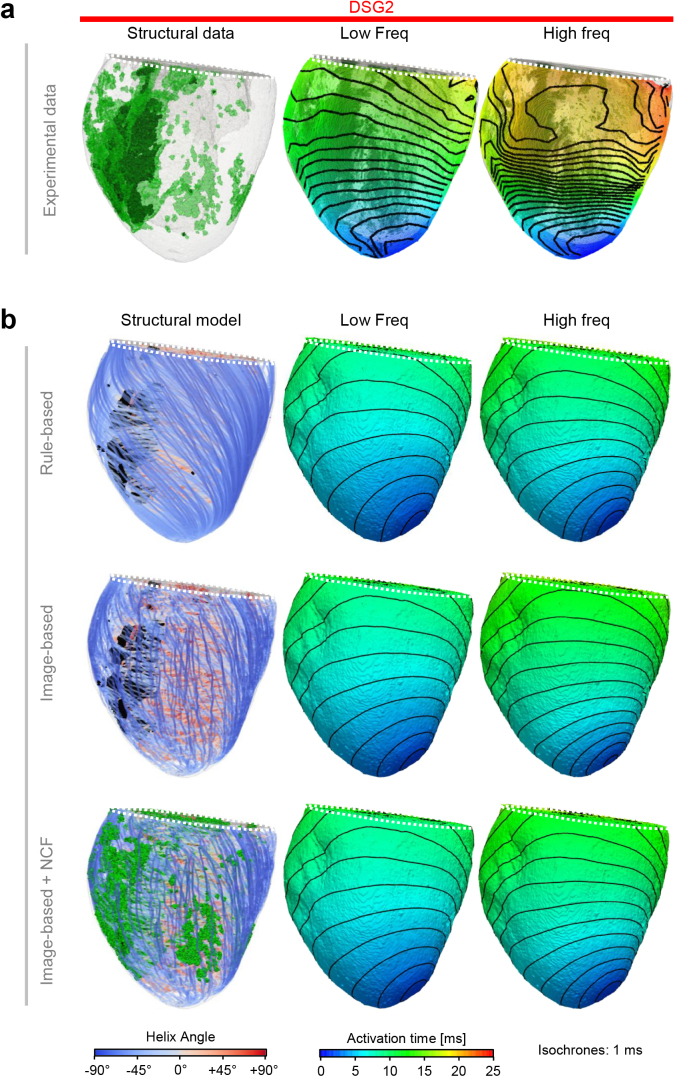
Integrated computational modelling of AP propagation. **a**: Electrical propagation upon apical pacing at LF (5 Hz) or HF (15 Hz) simulated by a functional model of electrical activity in one DSG2 heart. Structural information was integrated into a bi-ventricular geometrical model (spatial resolution: 100 μm) based on myocardium anatomy and compact fibrosis (CF) at three progressive levels: a theoretical distribution of cardiomyocytes (rule-based), real cardiomyocytes organization (image-based), and after adding non-compact fibrosis (NCF) distribution. The figure show the 3D activation maps of the last beat (after 10 pulses at LF and HF). Ventricular cavity orifice was shown in blue for better visualization. **b** Experimental activation maps by optical mapping on same experimental conditions (apex pacing at low and high frequency) were mapped on the same geometry.

Modeling was conducted at three different levels of personalization (Fig. 6b and Extended Data Fig. 9), beginning with personalized anatomical models (including nonconductive CF patches) combined with a standardized distribution of cardiomyocytes (rule-based fiber orientation), progressing to a more comprehensive scenario where the individually observed cardiomyocyte organization (image-based fiber orientation) was included, and finally a model where NCF was additionally integrated by defining a percentage of the mesh elements tagged as NCF as non-conducting tissue proportionally to the scattering signal intensity^22^.

We found that, irrespective of the anatomical accuracy, a simple reaction–diffusion-based AP propagation model can describe myocardial excitation and conduction in CTRL hearts and in DSG hearts at LF pacing. However, it fails to predict the pacing-rate dependency of conduction in DSG2 hearts, where only ≈3–4-ms prolongation of activation time is predicted at HF compared with LF (an increase of ≈25%), in contrast to the ≈10-ms (≈60%) prolongation observed experimentally in the same hearts (Fig. 6a and Extended Data Fig. 9).

This discrepancy indicates that integrating structural properties alone, even at high resolution, is not sufficient to explain the conduction anomalies revealed at HF pacing in DSG2 hearts without considering other features of electrophysiological remodeling.

### Pacing-rate dependency of electrophysiological remodeling in fibrotic regions

**Fig. 7:**
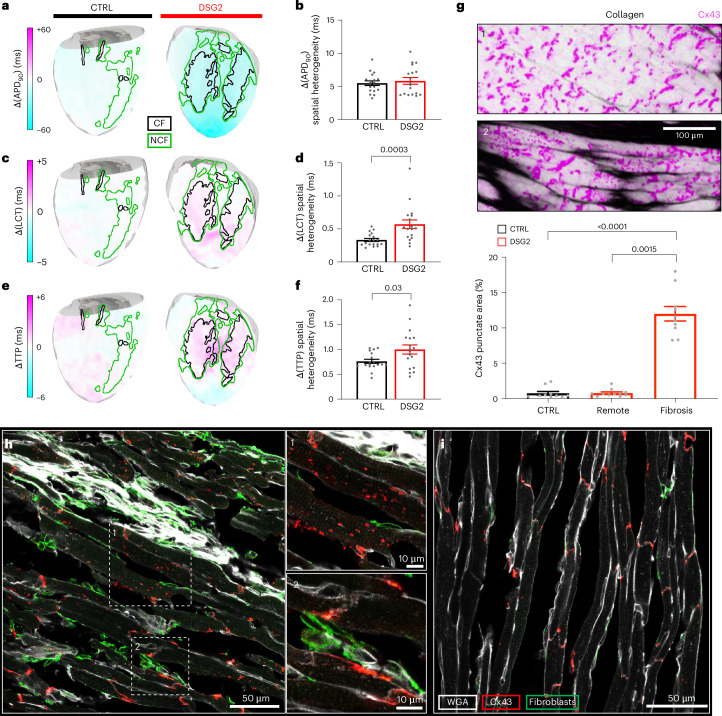
Morpho-functional registration between fibrosis and electrophysiological remodeling. **a**,**c**,**e**, Differential maps indicating variations in APD_90_ (**a**), local conduction time (LCT; **c**) and TTP (**e**) upon pacing at the apex at LF and HF (shown as HF minus LF), for one CTRL heart and one DSG2 heart. Functional maps are mapped onto the 3D mesh, and contours of CF and NCF areas are highlighted in black and green, respectively, for better visualization. **b**,**d**,**f**, Spatial heterogeneity (in ms) calculated from APD_90_ (**b**), LCT (**d**) and TTP (**f**) differential maps, measured from two angles to ensure mapping of the entire LV of each heart (two points per heart are reported). **g**, Quantification of Cx43 punctate area in remote (inset 1 from Fig. 5c) and fibrotic regions (inset 2) of DSG2, and compared with CTRL. Data are reported as means ± s.e.m. Data in **b**, **d** and **f** were collected from nine CTRL and nine DSG2 mouse hearts. A two-way repeated-measures ANOVA was performed with a Tukey post hoc comparison. Data in **g** were collected from two CTRL (ten randomly selected areas) and two DSG2 mouse hearts (ten randomly selected areas from remote and ten from fibrotic areas each). Data were analyzed using the Kruskal–Wallis test followed by Dunn’s multiple-comparisons test (two-sided). **h**,**i**, Immunofluorescence signal of cell membrane (WGA), Cx43 and PDGFR (a fibroblast marker) from a confocal reconstruction of a 10-µm cryosection (mid-ventricular portion) of a non-cleared DSG2 mouse heart, in proximity to a fibrotic region (**h**) and in a remote area (**i**). Inset 1: re-localization (internalization) of Cx43 in myocytes; inset 2: coupling between fibroblasts and myocytes via Cx43 (observed in four sections from two DSG2 hearts). Source data

Building on the simulation results, we aimed to explore relevant electrophysiological changes occurring in fibrotic areas when switching from LF to HF pacing rates by generating difference maps of APD_90_, local conduction time and TTP. Difference maps were then co-registered with structural data to correlate frequency-dependent electrophysiological differences with CF and NCF (Fig. 7). We found that CTRL and DSG2 hearts show a uniform reduction in APD_90_ when comparing HF to LF (Fig. 7a), with no significant difference in spatial heterogeneity between groups (Fig. 7b). These data invalidate the hypothesis that wavefront deformation in DSG2 is caused by overt differences in cardiomyocyte AP restitution curves in fibrotic and non-fibrotic tissue, which could otherwise alter local refractoriness and prevent conduction at HF. On the other hand, we found a higher spatial heterogeneity of differential local conduction time and TTP in DSG2 hearts with respect to CTRL (Fig. 7d,f): local conduction time was predominantly increased where the AP wave first reaches NCF/CF tissue areas (Fig. 7c), and these changes are spatially strongly correlated with the increase of TTP found in same area (Fig. 7e).

In line with these findings, we observed differences in Cx43 distribution near CF, showing more punctae compared with the remote area, where a linear morphology, preferentially aligned perpendicular to cell direction (as expected from intercalated discs), is preserved (Fig. 7g). To further explore the structural basis of this frequency-dependent electrophysiological remodeling, we performed Cx43, fibroblast and cell membrane staining in non-cleared CTRL (*N* = 2) and DSG2 (*N* = 2) hearts. Confocal imaging confirmed an enhanced presence of Cx43 punctae in proximity to fibrotic areas (Fig. 7h), compared with remote tissue (Fig. 7i), indicating a re-localization of Cx43 to the cytoplasmic compartment and cellular surfaces, as previously observed in the same cardiomyopathy^23^. This coincided with increased fibroblast presence in the same areas, with lateralized Cx43 colocalizing with cell membranes of both myocytes and fibroblasts.

Taken together, our findings indicate that the conduction disturbances observed at short coupling intervals are driven by localized conduction slowing at the interphase between myocardial and fibrotic tissue. In this region, the re-localization of Cx43 may impede myocyte-to-myocyte electrical coupling and enhance electrotonic coupling between myocytes and non-myocytes. These alterations in cellular coupling, together with the general reduction in sodium current amplitude observed in DSG2 mice^24,25^, which may be even more prominent in the presence of Cx43 punctae^26^, could induce the frequency-dependent conduction behaviors observed in fibrotic regions.

**Fig. 8:**
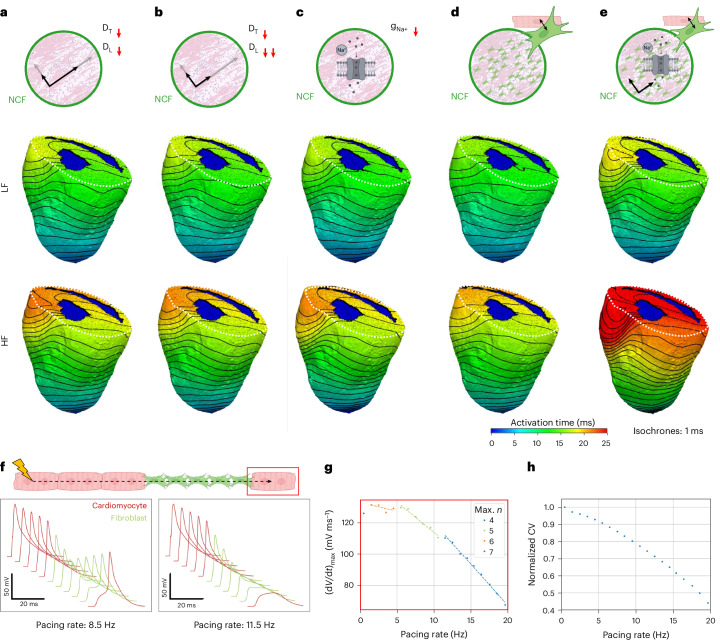
Simulation of local electrophysiological alterations in NCF. Simulations of apical pacing at LF and HF were performed on a structural model of a DSG2 heart, incorporating image-based fiber orientation and percolation-derived NCF (as in Fig. 6a, third row). Electrophysiological alterations were introduced within the fibrotic region (NCF). **a**, Reduced tissue conductivity by 75%, preserving normal anisotropy. **b**, Further reduced longitudinal conductivity achieving isotropy. **c**, Mimicking decreased sodium channel availability by reducing peak sodium conductance by 75%. **d**, Homogeneous addition of electrically coupled fibroblasts (1 myocyte connected to 12 fibroblasts). **e**, Combined condition: isotropic reduced conductivity, sodium current reduction and fibroblast coupling. **f**, Modeling of a 1D chain of electrotonically coupled myocytes and fibroblasts. Examples for successful (left; pacing rate of 8.5 Hz) and failing (right; pacing rate of 11.5 Hz) AP propagation between ventricular cardiomyocytes (red APs) via an insert of electrically non-excitable fibroblasts (green; here *n* = 5 cells). Signals are shifted for visualization; thus, axis labels do not indicate absolute values. **g**, Maximum number of insertable fibroblasts (max *n*) compatible with AP propagation up to the last cardiomyocyte (red rectangle) at different pacing rates. The maximum upstroke velocity ((d*V*/d*t*)_max_) of the final cardiomyocyte after the fibroblast chain is shown for each pacing rate, grouped by max *n* (colors). Dashed lines show the linear fit of upstroke velocities grouped by max *n*. **h**, Dependence of CV on pacing rate for a chain of *n* = 4 fibroblasts, inserted between cardiomyocytes. CV normalized to the CV at a pacing rate of 0.5 Hz. Source data

To test the validity of these hypotheses, we implemented a high-resolution modeling platform incorporating localized electrophysiological remodeling within NCF (Fig. 8a–e). We found that reducing myocyte-to-myocyte conductance—under both isotropic and anisotropic conduction conditions—did not remarkably affect conduction at high pacing frequencies. Only a modest slowing of conduction was observed, with ventricular activation delayed by approximately 4 ms at HF compared with LF. Similarly, a reduction in sodium current (≈5-ms prolongation) or an increase in myocyte/non-myocyte coupling (≈4-ms prolongation) alone failed to reproduce the wavefront deformation observed experimentally at HF. On the other hand, combining all these alterations resulted in a conduction behavior closely matching experimental findings at high pacing rates, with a prolongation in ventricular activation time of approximately 10 ms (comparable to delays observed experimentally).

**Extended Data Fig. 10:**
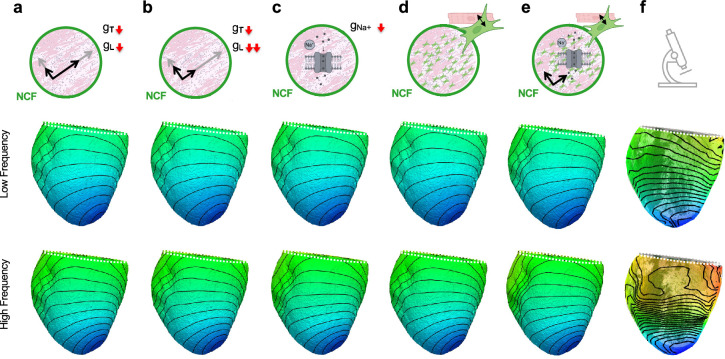
Simulation of Local Electrophysiological Alterations in Non-Compact Fibrosis. Simulations of apical pacing at low (LF) and high frequency (HF) were performed on a structural model of a DSG2 heart, incorporating image-based fiber orientation and percolation-derived NCF (as in Fig. S11, third row). Electrophysiological alterations were introduced within the fibrotic region. **a**: Reduced tissue conductivity to 25% of the healthy transverse value, preserving normal anisotropy. **b**: Same conductivity reduction as in A, but with additional reduction of longitudinal conductivity to achieve isotropy. **c**: decreased sodium availability by reducing peak sodium channel conductance to 25% of the healthy value. **d**: Presence of electrically coupled fibroblasts (1 myocyte connected to 12 fibroblasts). **e**: Combined condition with all three alterations: isotropic reduced conductivity, sodium current reduction, and fibroblast coupling. **f**: Experimental activation maps by optical mapping on same experimental conditions (apex pacing at low and high frequency) were mapped on the same geometry.

However, we found that this set of non-sample-specific electrophysiological alterations does not succeed in replicating the experimental data across all hearts (Extended Data Fig. 10; ≈4 ms of prolongation against ≈10 ms of prolongation measured experimentally), suggesting that additional mechanisms may be involved. For instance, the high density of non-myocytes in NCF would involve regions where the AP propagates passively through islands of non-myocytes at reduced speed^27^. To explore the potential involvement of this mechanism, we developed a one-dimensional (1D) model of a chain of coupled myocytes and fibroblasts, overcoming limitations of conventional bidomain models that homogenize cell-scale phenomena in 3D geometries. Our model demonstrates that passive AP propagation through a fibroblast insert between groups of myocytes fails to effectively activate downstream myocytes when increasing pacing rate (Fig. 8f). Moreover, the model predicts a reduction in conduction velocity and a decreased maximum upstroke velocity (d*V*/d*t*)_max_ with increasing pacing frequency (Fig. 8g,h)—all of which aligns fully with our experimental findings.

## Discussion

It is widely recognized that myocardial fibrosis is a key contributor to cardiac arrhythmias. Indeed, the presence of myocardial fibrosis has been shown to be a strong predictor of sudden cardiac death secondary to ventricular arrhythmias in patients^28^. However, the exact role and impact of fibrosis on arrhythmogenesis remain elusive. This complexity arises from the heterogeneous nature of fibrosis, encompassing diverse densities and architectural patterns, each potentially exerting distinct effects on cardiac excitation dynamics^29–31^. Furthermore, while cardiac scars have traditionally been viewed as nonconductive (even ‘dead’) tissue, fibrotic lesions are a highly dynamic tissue^32^, often contain strands or islands of surviving cardiomyocytes^3^ and a considerable number of non-myocytes^32^, which are known to potentially support electrotonic cross-talk with cardiomyocytes^27,33,34^. Therefore, elucidating the impact of fibrotic tissue on arrhythmogenesis necessitates a comprehensive approach that integrates detailed reconstructions of cardiac cellular and extracellular matrix structural organization with a thorough assessment of electrophysiological properties within and outside the fibrotic tissue environment.

The possibility of correlating electrophysiological behavior with cardiac structural remodeling at high spatial resolution has emerged as a valuable approach for elucidating the mechanisms linking arrhythmic risk to fibrotic architecture^35^. Following a similar methodological approach to Zhu et al.^36^, we combined a state-of-the-art tissue clearing approach^37^ with advanced optical microscopy techniques^38^ to co-register functional optical mapping data with 3D reconstructions of cardiomyocytes and fibrosis organization across entire mouse ventricles at an unprecedented spatial resolution.

To overcome the limitations of incomplete antibody diffusion in whole-mount SHIELD-cleared hearts^17^, we used a alternative approach: 3D reconstructions guided the selective mechanical sectioning and immunostaining of thick transversal slices of cleared tissue. The resulting structural data were used for comprehensive morphometric characterization of the hearts and to generate organ-specific integrated computational models of AP propagation.

The DSG2 mouse model used here has previously been shown to exhibit impaired cardiac function, as well as extensive myocardial fibrosis and altered distribution of junctional proteins, including Cx43 (ref. ^12^). Here, by co-registering functional and structural data, we observed that the regions of heterogeneous conduction of activation wavefronts in the LV free wall are near fibrotic patches, and they become overtly evident while pacing at HF, when local conduction is strikingly slowed down in the proximity of NCF/CF tissue. These data demonstrate a clear link between fibrotic remodeling and conduction velocity restitution. We simulated the effect of fibrosis on ventricular activation in the DSG2 model by using an image-based organ-specific computational model. We found that electrophysiological dysfunction cannot be fully predicted in silico, even when using high-resolution structural data of cardiomyocyte and collagen organization, in combination with a state-of-the-art method for simulating diffuse fibrosis^39^. Indeed, by comparing APD maps while pacing the heart at LF and HF, we found that APD at HF is shortened homogeneously across the myocardium, suggesting that refractoriness is unlikely to vary systematically across the ventricle, implying that the disruption of conduction is not caused by inexcitability of the fibrotic area. Instead, we observed an increase of TTP at HF with respect to LF pacing rates in the tissue regions where the activation wavefront reaches NCF/CF tissue.

These findings suggest that fibrotic remodeling within specific regions of the ventricular myocardium results in alterations to electrical properties of the tissue, effectively creating a low-pass filter for cardiac conduction. This low-pass filtering enables AP propagation through the fibrotic tissue at low heart rates, while attenuating or blocking conduction at higher heart rates.

A reduction of conduction velocity has previously been observed at the border of the infarct zone in a rabbit model of chronic myocardial infarction^40^. This slowed conduction may be attributed to the passive electrotonic load imposed on cardiomyocytes by connexin-coupled non-myocytes within the fibrotic tissue. These non-myocytes act as current sinks, thereby reducing the maximum upstroke velocity of the AP and consequently slowing down the rate of conduction^41,42^.

We propose that a similar mechanism underlies the low-pass filtering effect, observed in this investigation. Under physiological conditions, gap junctions are predominantly localized between adjacent cardiomyocytes at the intercalated discs, facilitating fast spread of excitation along the longitudinal axis of cardiomyocytes. In fibrotic regions of DSG2 hearts, redistribution of Cx43 as well as reduced sodium current impair myocyte–myocyte electrical coupling and slow conduction. In parallel, myocyte/non-myocyte electrotonic coupling increases the local current sink, leading to a local elevation in capacitance, slight resting membrane depolarization (as cardiac fibroblasts have an inherent membrane potential that is more depolarized than that of cardiomyocytes^43^) and reduced AP upstroke velocity. Consequently, the time constant of the equivalent electrical circuit increases, resulting in a slowing or termination of propagation at higher frequencies^5,6^.

Although a frequency dependence of conduction through fibrotic myocardium has been proposed in previous studies, those investigations were limited to in silico and/or in vitro models^7,8,29,30,44^. Our results establish this mechanism at the ex vivo whole-heart level, where a localized increase in current sink via heterocellular coupling can induce a functional block, ultimately leading to reentrant arrhythmias.

Taken together, our findings have several substantial implications. Firstly, the observed deformation of the wavefront at high stimulation rates could impair cardiac contractility, particularly during conditions requiring efficient pump function, such as exercise or stress. More critically, we demonstrate that this phenomenon plays a crucial role in the induction of a functional substrate for reentrant arrhythmias, such as triggered by ectopic beats. These findings underscore the paramount importance of characterizing conduction dynamics across a range of pacing frequencies, using dynamic pacing protocols.

This study further underscores that the electrical dysfunction associated with cardiac fibrosis, along with certain arrhythmogenic mechanisms, cannot be reliably predicted by models based solely on the structural organization of the myocardium and extracellular matrix. This remains true even when using spatial resolutions far exceeding the limits of current clinical imaging techniques, particularly when fibrotic tissue is modeled as electrically disconnected.

The results presented here emphasize that an accurate integration of electrophysiological remodeling within and around fibrotic areas into predictive models is essential to obtain reliable predictions. This prompts a reconsideration of modeling approaches relying exclusively on structural data (such as myocardial shape, cellular orientation and extracellular matrix), and on the assumption that cardiomyocytes and non-myocytes in the heart are electrically insulated from one another. Importantly, our findings indicate that this electrophysiological remodeling should also be personalized, as we found that models incorporating general electrophysiological remodeling sometimes fail to reliably reproduce experimental observations. One of the most prominent personalized approaches is the novel digital twin strategy^45^, which has recently also been implemented with personalized electrophysiological remodeling^46–48^. While these advances are hugely exciting, we propose that further efforts to incorporate cardiomyocyte/non-myocyte electrotonic coupling will improve the ability to predict arrhythmia susceptibility and to steer possible interventions in remodeled cardiac tissue^49,50^. Specifically, spatially heterogeneous changes in functional conduction (for example, gap junction density and distribution) or in cellular ionic properties (which govern excitability and recovery/restitution) may be impacted in a subject-specific manner. This variability between subjects may prove to be essential to accurately replicate experimentally observed frequency-dependent responses, in addition to the influence of individualized structural heterogeneities in anatomy, fibrosis and myofiber architecture.

This study highlights the importance of integrating functional and structural data at the whole-organ level to gain reliable insights into conduction disturbances in remodeled hearts. Given the distinct patterns of fibrosis localization in this mouse model, we were able to integrate 3D structural data with conventional 2D surface optical mapping on the LV free wall, generating valuable insights for this cardiomyopathy. In conditions with more diffuse morpho-functional abnormalities, the use of a panoramic optical mapping system^51,52^ may be beneficial to interrelate structural and functional data across both ventricles.

Moreover, although optical mapping is sufficient to assess electrical dynamics in small rodent hearts—which do not display appreciable transmural dynamics (Supplementary Fig. 7)—a volumetric optical mapping system would be advantageous in larger animals, such as rabbits or pigs. Using such an approach, transmural information could be extracted by leveraging the different tissue penetration depths of various excitation wavelengths, enabling multiplexed imaging across multiple myocardial layers^53^ and opening the possibility of unifying functional and structural data in animal models that more closely mimic human cardiac pathophysiology.

Additional important perturbations, such as altered calcium homeostasis^54,55^, could be investigated by enhancing this imaging framework through combined voltage and Ca²⁺ mapping. Moreover, integrating all these experimental data into an advanced modeling platform—based on a multi-domain approach that explicitly incorporates myocytes and non-myocytes in a 3D cellular context—would provide a powerful tool for refining predictive concepts in cardiac arrhythmogenesis. For example, this approach could be used to simulate the electrophysiological effects of partial gene delivery, as occurs in adeno-associated virus (AAV)-mediated therapies, where only a fraction of cardiomyocytes express the transgene^56,57^. By modulating key parameters such as sodium current amplitude and Cx43-mediated coupling within a spatially heterogeneous, cellular-scale framework, the model could support in silico predictions of anti-arrhythmic versus pro-arrhythmic outcomes in gene therapy scenarios. Such approaches would not only enhance our predictive capabilities, but also inform personalized therapeutic strategies, including pharmacological interventions and the rational design of next-generation gene therapy approaches^58,59^.

## Methods

### Animal model

For the morpho-functional investigation, we used nine male *Dsg2*^mut/mut^ mice (DSG2)^12^ and nine male littermate controls (CTRL) aged between 7 and 13 months. For structural investigation by confocal microscopy, four additional mice (two DSG2 and two CTRL mice, aged between 6 and 10 months) were used. Mice were housed in an animal facility with a regular 12-h light–dark cycle with unrestricted access to food and water. All experimental procedures performed were approved by the local ethics committee of the Ministry of Health (authorization numbers 175/2002A, 408/2018PR and 944/2018PR) in compliance with Italian animal welfare law (law n.116/1992 and subsequent modifications) and are compatible with the guidelines stated in Directive 2010/63/EU of the European Parliament on the protection of animals used for scientific purposes. Mice were housed with 12-h light–dark cycles, controlled humidity and constant ambient temperature. All procedures were performed by trained personnel with documented formal training and previous experience in experimental animal handling and care.

### Optical mapping

CTRL and DSG2 mice were heparinized by intraperitoneal injection (0.1 ml, 5,000 units per ml) and anesthetized by 5% inhaled isoflurane. The heart was excised with the mouse in deep terminal anesthesia, immediately cannulated at the aorta, and perfused with a Krebs–Henseleit buffer solution (containing 120 mM NaCl, 5 mM KCl, 2 mM Mg_2_SO_4_^−^7H_2_O, 20 mM NaHCO_3_, 1.2 mM NaH_2_PO_4_-H_2_O, 10 mM glucose). The solution at 36 °C ± 1 °C was at pH 7.3 when equilibrated with carbogen (5% CO_2_/95% O_2_). Cardiac contractions were inhibited by addition of 10 μM (±) blebbistatin to the solution. The cannulated heart was placed in a custom-built optical mapping chamber (horizontal Langendorff system) and perfused at a constant flow of 2.5 ml min^−1^ at 36 °C ± 1 °C. After stabilization of the electrocardiogram, typically within a few minutes, 1 ml of perfusion solution containing the voltage-sensitive dye (VSD; di-4-ANBDQPQ; 8 μg ml^−1^, University of Connecticut Health Center) was injected into the aorta over the course of 1 min. Whole mouse hearts were illuminated in a wide-field configuration using a ×2 objective (TL2x-SAP, Thorlabs) and a light-emitting diode operating at wavelengths centered around 625 nm (M625L3, Thorlabs) projecting through a band-pass filter at 640/40 nm (FF01- 640/40-25, Semrock). The heart was illuminated with a maximum intensity of 1 mW mm^2^. A dichroic beam splitter (FF685-Di02- 25 × 36, Semrock), followed by a band-pass filter at 775/140 nm (FF01- 775/140-25, Semrock), collected the VSD-emitted fluorescence. A ×20 objective (LD Plan-Neofluar ×20/0.4 M27, Carl Zeiss Microscopy) was used to focus the fluorescence of a FOV of about 10 × 10 mm in the sample plane on the central portion (128 × 128 pixels; resulting in a per-pixel sample size of 78 μm × 78 μm) of the sensor of an sCMOS camera (ORCA-Flash 4.0, Hamamatsu Photonics), operating at a frame rate of 1 kHz (1 ms actual exposure time). For electrical stimulation, a tungsten bipolar electrode (WE5ST30.5B10, Science Products), connected to an isolated constant voltage stimulator (DS2A, Digitimer), was used to deliver voltage pulses at the apex. Optical recordings of the LV free wall were collected while pacing the heart with 15 pulses at increasing frequencies: 5, 6, 8, 10, 12 and 15 Hz. In the paper, LF refers to recordings obtained while pacing at 5 Hz, while HF recordings represent the maximum frequency the heart was able to follow in a 1:1 manner (usually 10 to 15 Hz).

Susceptibility to cardiac arrhythmias was assessed by electrically pacing the heart at the right ventricular outflow tract. The heart was paced for 4 × 10 s with pacing intervals equal to 1.7 times the sinus rhythm interval, thereby pacing at varying delays after sinus beats.

Arrhythmia inducibility was quantified from optical recordings by assessing the number of arrhythmic events, provoked by an electrical stimulus that occurred within 75 ms from the onset of the depolarization of the preceding sinus beat.

### Optical mapping analysis

All programs for optical mapping data acquisition and analysis were developed with LabVIEW 2015 (National Instruments, version 15.0, 64 bit). To process obtained images, the mean baseline was first subtracted and then the frame was normalized on a pixel-by-pixel basis, yielding a percentage change in fluorescence over time (∆*F/F*_0_). Next, ∆*F/F*_0_ signals were inverted and a binning operation was performed on areas of 4 × 4 pixels to reduce noise, generating parameter maps with a spatial resolution of 312 μm. Average AP kinetics were analyzed by assessing the time between the onset of the AP and the peak (TTP), and to 50%, 70% and 90% of recovery of resting fluorescence signal intensity (APD_50_, APD_70_ and APD_90_, respectively). Values were obtained across the entire visible surface of the left ventricle and values for individual pixels were averaged to obtain one value per ventricle for interindividual comparisons.

Conduction velocity was calculated using activation maps, after a spatial binning of 4 × 4 pixels using a multi-vector approach. A seed reference pixel was arbitrarily chosen, and the cross-correlation of the fluorescence trace was calculated pixel by pixel to estimate the temporal shift among every pixel. Sequential frames were then collapsed into one frame where each pixel corresponds to the delay in time of activation with respect to a user-defined initial time. Deformation of the activation wavefront was assessed by calculating the standard deviation of conduction vector angles.

To generate the differential maps of APD_90_, local conduction time and TTP, the map of each parameter while pacing at LF was subtracted from the map obtained while pacing at HF, generating new maps where each individual pixel shows the difference between low and high frequencies. To generate these differential maps, optical mapping was performed twice per heart to ensure mapping of the entire LV surface. Local conduction time was determined by calculating the delay between the activation time of adjacent pixels, thereby revealing localized differences in conduction velocity, if present. To quantify the spatial heterogeneity of the individual differential maps, the standard deviation across all pixels was calculated.

### Tissue clearing

After optical mapping, hearts were immediately Langendorff-perfused with 4% paraformaldehyde in phosphate-buffered saline (PBS) solution and incubated in the same solution overnight at 4 °C. Next, the atria were removed before samples were optically cleared using a cardiac-modified SHIELD protocol^17^. Hearts were washed in PBS three times for 1 h each at 4 °C. Then, the hearts were incubated in 20 ml of SHIELD-OFF solution (consisting of 25% double-distilled H_2_O, 25% LifeCanvas Technologies SHIELD Buffer solution, 50% LifeCanvas Technologies SHIELD Epoxy solution) at 4 °C for 6 days. Thereafter, hearts were incubated in 20 ml of LifeCanvas Technologies SHIELD-ON solution at 37 °C for 24 h, followed by SHIELD clearing solution (300 mM sodium dodecyl sulfate, 10 mM boric acid, 100 mM sodium sulfite; pH 9) at 37 °C. The clearing solution was refreshed twice a week for 5 months. All the steps were performed while rocking the samples at 40 rpm (SSM3 Gyro Rocker). Transparent hearts were then washed twice in PBS for 24 h, and finally incubated in 10 ml of refractive index matching solution (EasyIndex, LifeCanvas Technologies) at room temperature for 2 days before imaging.

### MesoSPIM and whole-ventricle reconstruction

Whole ventricles were reconstructed in 3D space, using a custom-made mesoscale selective plane-illumination (mesoSPIM) microscope^60^ (Supplementary Fig. 2). All microscope hardware components were automatically controlled by a dedicated software developed in LabView 2012 (National Instruments, version 12.0f3 at 32 bit).

The microscope was equipped with two excitation arms (left and right) consisting of a laser combiner (L4CC-CSB-1311 with fast switching dual-output module MDL-FSTM, Oxxius) operating at 638 nm as a light source, collimated on a tunable lens translating the focal line of the light sheet (that is, the ‘waist’) along the horizontal (*x*) axis of the FOV. A high-frequency galvanometric mirror generated the light sheet in the *XY* plane by sweeping the light beam along the vertical (*y*) axis, while an excitation lens focused the excitation beam on the sample. A ×2 telecentric lens (TM42-10M-20-75, Lenstation) and a high-performance back-illuminated sCMOS sensor (Kinetix Scientific CMOS Camera, Teledyne Photometrics) acquired a square FOV (10.4-mm side lengths, with a pixel size of 3.25 μm), sufficient to image optical cross-sections of the entire mouse ventricles with negligible field-curvature distortion.

Both the left and right excitation arms generated a sheet of light. To optically cut an internal plane of the sample, the system initially generated a sheet of light from the left arm, shifting the focus line from *x*_min_ along the *x* axis (defining the FOV width from *x*_min_ to *x*_max_, where *x*_center_ is the central part of the FOV, that is, in the middle of *x*_min_ and *x*_max_). Once reaching the *x*_center_ position, the laser combiner rapidly (<5 ms) switched the excitation light to the second output enabling the right arm illumination, programmed to translate the focus from *x*_center_ to *x*_max_, that is, toward the right arm. The camera’s rolling shutter, 64 lines wide (about 208 μm), was synchronized with the sliding of the focal line of the illuminating light sheet, which takes 500 ms to travel through the entire FOV. Finally, the system recovers the initial waist position in 20 ms, ready for the next scan. The resulting optical section had a full width at half maximum of 6.7 μm (Supplementary Fig. 3), with a total time between the excitation of two consecutive optical planes of 520 ms.

For sample stabilization, the base of the heart was glued to a metal support using a commercial glue (Super Attak Precision, Loctite) without damaging the ventricular tissue. The sample was placed into a quartz cuvette (*n* = 1.46, UQ-204, Portmann Instruments) filled with a refractive index matching solution (EasyIndex, LifeCanvas Technologies, *n* = 1.46) and mounted with magnets on the support structure. This support was equipped with two manual translators (Physik Instrumente) for positioning the sample in the FOV *x–y* plane, a motorized translator for movements along the *z* axis during 3D data acquisition (Physik Instrumente, M-122.2DD) and a rotational translator (Physik Instrumente, M-116.DG). The whole block was moved downward until the sample cuvette was immersed inside a second, larger quartz cuvette (UQ-753, Portmann Instruments), filled with 68% 2,2′-thiodiethanol in PBS^61^ for homogeneity of the refractive index.

Tomographic reconstructions were obtained by moving the sample along the *z* axis at a constant speed of 6 μm s^−1^ and with an image plane acquisition frequency of 1.92 Hz (equal to 1/520 ms), resulting in a *z* step of about 3.1 μm. The motorized translation system moved the sample along the *z* axis to its starting position at the end of each whole-ventricle tomography; then, the rotational motor rotated the sample around the *y* axis by +90°, and tomography was repeated for a total of four acquisitions at 0°, 90°, 180° and 270° of rotation. This protocol was repeated two times: first, the sample was illuminated with a power of 1 mW, collecting the scattering signal; then, the sample was illuminated with a power of 30 mW, filtering the emission with a stop-band filter (NF03-405/488/561/635E-25, StopLine quad-notch filter, Semrock). We thus obtained eight tomographies, all represented by images at 16 bit and with a voxel size of 3.25 μm × 3.25 μm × 3.1 μm in *xyz* space. The impact of the incident direction of excitation light on the intensity of the detected scattering signal was also evaluated: two tomographies of the same sample—performed one at 0° and one at 90° rotation around the *y* axis—were realigned in 3D and the scattering signals compared (Supplementary Fig. 8).

After completion of the imaging protocol, preprocessing steps were applied to generate reliable near-isotropic dual-channel tomograms for each sample (Supplementary Fig. 9). Specifically, the best front/back pair (the two options are the front/back acquisitions at 0°/180° or those at 180°/270°) was selected by a user-defined qualitative check of the images (Supplementary Fig. 9a). The corresponding 3D images were resampled to an isotropic voxel size of 6 μm × 6 μm × 6 μm using bilinear interpolation and then converted to 8 bit using ImageJ software. This step reduced the file size (from about 40 GB to 3.5 GB for each single-channel partial tomogram) and intrinsically performed a 3D blurring operation that removed signal noise. Fluorescence and scattering signals were 3D registered to correct microscopic misalignments between channels using the Fijiyama plugin of ImageJ^62^ (Supplementary Fig. 9b), before the image pair was fused (Supplementary Fig. 9c) by means of the Huygens software (Huygens Professional version 19.04, Scientific Volume Imaging). Whole-ventricle reconstructions were then manually aligned to the reference system defined in the Standardized Myocardial Segmentation and Nomenclature for Tomographic Imaging of the Heart^63^ by the American Heart Association (Supplementary Fig. 9d). Supplementary Video 1 shows the final tomographic reconstruction of a DSG2 mouse heart after the preprocessing. Supplementary Video 2 shows a 3D rendering of a DSG2 heart reconstruction.

### Histological analysis of cleared tissue

Three cleared CTRL hearts and one cleared DSG2 heart were used for histological analyses (Supplementary Fig. 2). Short-axis cross-sectioning of the specimens from mid-ventricular to apical levels (bread-loafing) was performed. Samples were fixed again in 10% neutral buffered formalin at 4 °C overnight and then embedded in paraffin. Serial sections (4 µm thick) were stained with H&E or Masson’s trichrome, and were digitally scanned at an original magnification of ×400 using the Aperio AT2 platform (Leica Biosystems) into whole-slide digital images. Each SVS format file was imported into HALO digital imaging analysis software, version 3.6.4134 (Indica Labs).

### Two-photon imaging of cleared tissue

Four cleared and mesoSPIM-reconstructed samples (two CTRL hearts and two DSG2 hearts) were additionally imaged at subcellular resolution for Cx43, cellular membrane outlines and collagen detection. Hearts were washed in PBS at room temperature for 3 days, changing the solution every 24 h, and were then embedded in 3.5% agarose in PBS. Agarose-embedded samples were cut into 300-μm-thick slices using a vibratome (model 7000smz 2, Campden Instruments), operating at a blade vibration frequency of 60 Hz and a forward speed of 0.05 mm s^−1^. For each heart, three slices were stained: one each from near-apical, equatorial and near-basal regions of the heart. Slices were incubated in PBS + 1% Triton X-100 (PBS-T 1×) for 24 h before staining.

For labeling, slices were incubated with 1:200 anti-Cx43 antibody (C6219, produced in rabbit, Sigma-Aldrich) at 37 °C for 3 days in PBS-T 1×. The samples were then washed in PBS for 6 h and incubated with 1:100 CNA35-eGFP^64^, 1:100 WGA (WGA–Alexa Fluor 594, W11262, Thermo Fisher Scientific) and 1:200 secondary antibody (goat anti-rabbit conjugated to Alexa Fluor 647, ab150079, Abcam) for 2 days at room temperature in PBS-T 1×. The collagen probe CNA35-eGFP recognizes the triple helix of a variety of fibril-forming collagens including type I, III and IV^64^. The plasmid encoding pET28a-EGFP-CNA35 was a gift from M. Merkx (Addgene plasmid no. 61603). The protein was produced in *Escherichia coli* and eluted by a solution containing 150 mM imidazole, 20 mM Tris-HCl (pH 7.9) and 500 mM NaCl. The probe was then concentrated (15.94 mg ml^−1^) and the buffer was exchanged to 50 mM Tris-HCl (pH 8.0). After incubation, slices were washed in PBS for 24 h at room temperature and mounted in a custom-made imaging device.

For imaging, each sample was placed on a cover glass (75 mm × 25 mm) together with a thickness-matched holed spacer (74 mm × 24 mm), carefully covered with a 250-μm-thick quartz glass matching the refractive index of the sample, and the chamber was sealed with a bicomponent glue (Twinsil speed, Picodent). The sandwich was then filled via an access port in the spacer with 1.5–2 ml of EasyIndex using a 1.0-ml syringe and a 0.45-mm needle.

These slices were imaged with a two-photon microscope (TCS SP8 DIVE, Leica Microsystems) using a water-immersion objective (HC IRAPO L ×25/1,00 W motCORR, Leica). The system is equipped with a tunable laser line (680–1,300 nm) and an additional fixed line at 1,045 nm. The two-photon excited fluorescence can be recorded on four non-descanned spectral detectors (two PMT and two ultrasensitive hybrid detectors, HyD). Image acquisition was performed using excitation wavelengths of 920 nm and 1,045 nm, whereas the detection windows were set to 494–550 nm for CNA35-eGFP (by PMT), 595–637 nm for WGA–Alexa Fluor 594 (by HyD) and 671–696 nm for Cx43-Alexa Fluor 647 (by HyD). The whole slices were imaged in a mosaic scanning mode (Leica LAS-X Navigator). Serial adjacent *z*-stacks were acquired with 10% *x*–*y* overlap and subsequently stitched together (*xy* pixel size, 0.86 μm; *z*-step size, 2 μm).

### Confocal imaging of non-cleared tissue

A total of four hearts were used for structural analysis by immunostaining and confocal microscopy, including two DSG2 hearts (8 and 10 months old) and two CTRL hearts (6 and 8 months old). After excision, the hearts were perfused through the aorta, first with 20 ml of PBS, followed by 15 ml of 4% PFA. Hearts were then fixed overnight at room temperature using 4% PFA. After fixation, hearts were washed in PBS three times for 1 h each.

The apex from each heart was cut off and the hearts were embedded in Tissue-Tek O.C.T. compound (Sakura Finetek). These samples were frozen using liquid nitrogen and stored at −80 °C until further use. Myocardial tissue was cryo-sectioned into 10-µm slices using a Leica CM3050 S cryostat operated at −20 °C (Leica Biosystems). Cut sections were collected on glass slides (VWR SuperFrost Plus) and stored frozen until further processing.

For immunofluorescence labeling, the samples were thawed and fixed with 2% formaldehyde solution for 15 min at room temperature. Slices were washed with PBS solution and then blocked and permeabilized with 0.5% Triton X-100, 5% donkey serum and 5% BSA in PBS (30 min, room temperature). Primary antibodies were added, and the samples were incubated in a humidified chamber at 4 °C overnight (rabbit anti-Cx43 at a 1:500 dilution, Abcam, ab11370; and goat anti-PDGFR-alpha at a 1:200 dilution, Novus Biologicals, AF1062-SP). Then the samples were washed with PBS containing 0.1% Tween and incubated with the secondary antibodies and WGA at a 1:1,000 dilution for 2 h at room temperature (donkey anti-rabbit Alexa Fluor 594, Abcam, ab150064; donkey anti-goat Alexa Fluor 488, Abcam, Cab150129; WGA–Alexa Fluor 555, Thermo Fisher Scientific). Next, DAPI was added at a dilution of 1:1,000 for 15 min at room temperature (D1306, Thermo Fisher Scientific). After final washing steps, the slides were drained before embedding the samples with PermaFluor mounting medium (Fisher Scientific) and covering them with a no. 1.5 coverslip. Samples were cured overnight at 4 °C. The next day, the samples were sealed using clear nail polish and stored at 4 °C until imaging.

All samples were imaged on an inverted laser-scanning confocal microscope (TCS SP8 X; Leica Microsystems). An overview image was acquired at low resolution in tile-scanning mode using a multi-immersion objective (HC PL APO CS2 ×20/0.75) with water. Higher-resolution *z*-stacks were recorded using a water-immersion objective (HC PL APO ×40/1.10 W CORR CS2) in areas of interest. The *x*–*y* pixel size was 68 nm, the *z*-step size was 840 nm. DAPI was excited with a 405-nm diode laser and the fluorescence signals were recorded with a photomultiplier tube (detection window, 415–479 nm). The other fluorophores were excited using lines from a white-light laser, and the signals were recorded on hybrid detectors operated in photon counting mode. The following lines and detection windows were used: Alexa Fluor 488—excitation 499 nm/detection 508–546 nm; Alexa Fluor 555—excitation 553 nm/detection 560–588 nm; Alexa Fluor 594—excitation 594 nm/detection 603–643 nm.

### Registration between two-photon and mesoSPIM sections

The distribution of collagen in two-photon images was compared with that obtained from the scattering signal in the mesoSPIM dataset. For each physical slice, a virtual cross-sectional plane was extracted from the mesoSPIM-based tomogram at the corresponding anatomical location along the apex-to-base axis of the heart (‘virtual 1’ in Extended Data Fig. 6a). A 3D rigid rotation was applied to a downsampled version of the tomogram to optimize the spatial alignment between the physical section and the virtual slice (‘Virtual 2’ in Extended Data Fig. 6a), thereby correcting for the inclination of the physical cut and enabling the virtual extraction of a corresponding 300-µm-thick section.

To quantify the reliability of fibrosis segmentation by scattering signal (performed on 3D mesoSPIM data with an isotropic voxel size of 20 µm), we first investigated the correlation between the scattering signal in the mesoSPIM data, and the collagen distribution reconstructed via two-photon microscopy. To do this, it was necessary to correct for deformations induced in cleared tissue slices by tissue processing (including physical sectioning, immunohistochemistry, tile-based two-photon microscopy and 3D stitching). To this end, two-photon images were registered to the corresponding mesoSPIM sections by applying an elastic warping transformation (Extended Data Fig. 6b), guided by manually selected fiducial points based on myocardial features (autofluorescence in mesoSPIM data and WGA signal in two-photon images), enabling a qualitative assessment of the reliability of the registration pipeline, as well as the sensitivity of the scattering signal in detecting collagen (Extended Data Fig. 6b,c). Despite the diffuse and highly heterogeneous nature of the scattering signal, as well as the different resolutions of the imaging modalities, spatial correlation between the mesoSPIM-derived scattering signal and the collagen signal obtained from two-photon microscopy was satisfactory, as quantitatively assessed using the DICE similarity coefficient, resulting in a value of 0.791 ± 0.026 for collagen segmentation (using the same threshold parameters applied in the 3D fibrosis reconstruction; Supplementary Fig. 10), normalized against the accuracy of myocardial tissue registration, which achieved a DICE score of 0.901 ± 0.046.

### Image segmentation and anatomical models

The 3D anatomies of CTRL and DSG2 mouse hearts were reconstructed and investigated by segmenting regions of healthy myocardium (MYO), CF and NCF by tracking fluorescence (FLUO) and scattering (SCATT) signals, respectively.

First, mesoSPIM tomograms were downscaled to an isotropic voxel size of 20 μm using bilinear interpolation in ImageJ, to reduce computational load during segmentation and to filter out irregularities in the scattering channel. The 3D segmentation process was performed with Seg3D, loading FLUO and SCATT channels separately. Channels were normalized and summed to create the virtual ‘tissue’ channel, simulating the nonspecific signal coming from the entire organ. The organ anatomy was then segmented by T_ch_, through a combination of threshold-based, Boolean and 3D morphological operators. In detail, the non-background volume was segmented by manually placing seeds in the ventricles, taking advantage of automatic threshold-based segmentation in Seg3D. Artifacts of the automatic process, such as islands of voxels in the background segmented as tissue (T), were corrected by applying a connected component operator (placing seeds in the ventricular myocardium), followed by manual inspection/correction. Surfaces were smoothed by applying a sequence of morphological operators (size of filter kernel expressed in pixel): erode (kernel = 1 pixel), dilate (2), erode (2), dilate (2). Subsequently, a connected component operator was applied again to the resulting segmentation, ensuring the tagging of only one connected volume as T.

The atrioventricular separation plane (AVS) was modeled by a 3D circular slice (S_circ_) with a thickness of 200 μm, to exclude any remaining atrial tissue and the area affected by glue during the imaging procedure. In detail, three points were manually selected in Seg3D, preserving as much ventricular tissue as possible, and a custom Python script generated S_circ_ from their coordinates. A sequence of logical and morphological operators was used to extract AVS, ventricular tissue (V) and background (B) volumes:

- AVS = T AND S_circ_,
- V = connected component of (T – AVS), seeds in ventricles,
- B = NOT(V),
- T = V OR AVS,

where T was the final segmentation of the overall ventricular anatomy.

Then, using a combination of logical and connected component operators applied to V, B, T and AVS, we extracted the segmentation of ventricular chambers (left, L_ch_, and right, R_ch_) and main coronary arteries and veins (‘vessels’, V_ess_). Each 3D segmentation was tagged differently, and no overlapping voxels were present.

After completely segmenting the organ morphology, regions of CF and NCF were segmented by SCATT.

In detail, CF was segmented with a thresholding on SCATT (Supplementary Fig. 10a). We selected the optimal threshold by involving the discontinuities in the fluorescence signal, where MYO is expected to be replaced by a dense fibrotic scar^65^. We found the best results with a threshold of 10, at which the SCATT signal filled the voids present within the myocardial tissue in the fluorescence channel, without overlap (Supplementary Fig. 10b). This method was then validated by comparing the resulting segmentation with two-photon reconstructions and Masson’s trichrome-stained histological sections of slices of the same hearts.

In contrast, NCF couldn’t be segmented using a visual approach, because collagen is distributed diffusely inside the MYO, resulting in areas where both MYO and collagen are present within the sub-volumes of imaging voxels. We first identified a unique threshold for each CTRL heart to segment all the areas where an excess accumulation of collagen (EXC) was present.

Thresholds were selected to obtain a percentage of volume with EXC in line with the percentage expected in the ventricular MYO of healthy mice (around 2.5% (refs. ^66,67^)), obtaining a mean threshold of 6.4 ± 1.1 (Supplementary Table 1). This threshold was applied to all samples (CTRL and DSG2) to segment EXC area, and the resulting NCF = EXC − CF.

Finally, CF and NCF segmentations were tagged separately and included in the anatomical T segmentations obtained by fluorescence.

### Anatomical investigation of myocardium and fibrosis

An anatomical investigation of myocardium and fibrosis was performed for each sample on 3D segmentations. Volumes of T, L_ch_/R_ch_ and CF/NCF were calculated by multiplying segmentations volumes (as the number of voxels) with the volume of a single voxel in mm^3^, that is, 0.02 mm^3 ^= 0.008 × 10^−3^ mm^3^. Left chamber dilation was quantified with the ratio between L_ch_ and T volumes.

Anatomical measurements of L_ch_ wall thickness were based on the American Heart Association Standardized Myocardial Segmentation and Nomenclature for Tomographic Imaging of the Heart (Supplementary Fig. 11).

The heart was divided into three equally thick portions, perpendicular to the long axis of the heart: apical (from the apical end of the LV chamber), mid-cavity (the central part of the ventricular walls, which include the bulk of papillary muscle tissue and septum) and basal (the remaining part up to the AVS). L_ch_ wall thickness (including both free wall and septum) was then measured in the central position of each of these portions. The basal and mid-cavity segments were measured with six endocardium-perpendicular lines each (B1, B2, B3, B4, B5 and B6, and M1, M2, M3, M4, M5 and M6, respectively) 60° apart, and the apical segment with four lines (A1, A2, A3 and A4), 90 degrees apart (Supplementary Fig. 11).

The percentage of CF and NCF inside each sample was evaluated by segmentation volumes, that is, 100 × (CF/T) and 100 × (NCF/T) respectively.

The radial (transmural) distribution of fibrosis of L_ch_ was analyzed with a custom analysis tool developed in Python 3.8. The software is designed to exploit the myocardial segmentation as a reference and to analyze the spatial distribution of any other segmentation, mapping the result on a 2D model of a transversal plane. The main steps are explained below.

First, the sample was automatically centered into the virtual volume that included it by evaluating the center of mass of the fluorescence signal (that is, the barycenter of the entire myocardium) and translating the data accordingly (Supplementary Fig. 12a). Later, the central transverse plane (halfway through the volume) of the segmentation was extracted to automatically detect the surfaces of the endocardium and epicardium: pixels placed on circles with incrementing radii were collected; the smallest and largest circles with any nonzero voxels were found; the correspondent radii (*r* and *R* respectively) defined the position of endocardial and epicardial surfaces, respectively (Supplementary Fig. 12b). This represents the normalized shape of the transversal section that maps the result of the spatial quantization. The content of CF and NCF segmentations was averaged along the long axis of the left ventricle creating a 2D 8-bit average transversal view (Supplementary Fig. 12c), where the spatial quantization was performed. Specifically, a radial (and angular) quantization was performed, splitting the image into 16 circles (and slices) with incrementing radii (and equally-spaced angles; Supplementary Fig. 12d), creating a 2D grid composed by ‘sector’ as the space within a wedge, bounded by two circular segments. Inside each sector, pixel values were summed, thus generating a universal map between hearts (16 radii × 16 circles).

Average maps were generated to compare CTRL and DSG2 samples. First, average epicardium and endocardium radii were calculated as given by equation (1):

1 $$\bar{r}=\frac{{\sum }_{i}{r}_{i}}{n};\bar{R}=\frac{{\sum }_{i}{R}_{i}}{n}$$

where *n* is the number of samples to average. Each sample’s map was spatially normalized between the averaged $\bar{r}$ and $\bar{R}$ radii, and sectors of normalized maps were averaged.

### Geometrical model generation

Virtual models of biventricular anatomy were generated from 3D segmentations for a subset of samples (two CTRL, four DSG2). In detail, for each case, the external segmentation surface was extracted from Seg3D (https://sci.utah.edu/2011/02/17/seg3d-2-0/) as a VTK iso-surface mesh and converted to an unstructured volumetric tetrahedral mesh (tetmesh) with an average element edge length of around 100 μm using fTetWild software^68^ (from https://wildmeshing.github.io/). Segmentations of internal structures were then tagged separately in the following order (from outer to inner surfaces): MYO, L_ch_ and R_ch_, NCF, CF, V_ess_, AVS. Supplementary Video 3 shows a 3D tetmesh of a DSG2 heart, where MYO is shown in transparent gray, CF in red and NCF in green. Meshtool (https://bitbucket.org/aneic/meshtool/src/master/) was used for tetmesh manipulation. ParaView (v5.12, KitWare) was used for tetmesh visualization.

### Detection of 3D cardiomyocyte orientation and virtual fiber direction model

We reconstructed the cellular orientation in sub-volumes *p* of 16 × 16 × 16 voxels in the fluorescence channel, resulting in a spatial resolution of 96 μm × 96 μm × 96 μm. For each *p*, local (voxel size) image gradient structure tensors *S* were first computed as given by equation (2):

2 $$S=\nabla I\nabla {I}^{T}* {g}_{{\sigma }_{s}}=\left(\begin{array}{rcl}{I}_{x}^{2}* {g}_{x} & \,\,\,\,{I}_{x}{I}_{y}* {g}_{y} & \,\,\,{I}_{x}{I}_{z}* {g}_{z}\\ {I}_{y}{I}_{x}* {g}_{x} & \,{I}_{y}^{2}* {g}_{y} & \,\,\,{I}_{y}{I}_{z}* {g}_{z}\\ {I}_{z}{I}_{x}* {g}_{x} & \,\,\,\,{I}_{z}{I}_{y}* {g}_{y} & \,\,\,{I}_{z}^{2}* {g}_{z}\end{array}\right)$$

where the first-order spatial derivates *I*_*x*_, *I*_*y*_ and *I*_*z*_ were estimated using second-order accurate central differences, whereas *g*_*x*_, *g*_*y*_ and *g*_*z*_ represent Gaussian smoothing filters. Local *S* elements were then averaged to extract the mean 3D tensor $\bar{S}$ and the spectral decomposition reported below was performed as given by equation (3):

3 $$\bar{S}=\left(\begin{array}{rcl}{\bar{S}}_{{xx}} & {\bar{S}}_{{yx}} & {\bar{S}}_{{zx}}\\ {\bar{S}}_{{yx}} & {\bar{S}}_{{yy}} & {\bar{S}}_{{zy}}\\ {\bar{S}}_{{zx}} & {\bar{S}}_{{yz}} & {\bar{S}}_{{zz}}\end{array}\right)=\left(\begin{array}{rcl}\vdots & \vdots & \vdots \\ {{\bf{v}}}_{1} & {{\bf{v}}}_{2} & {{\bf{v}}}_{3}\\ \vdots & \vdots & \vdots \end{array}\right)\left(\begin{array}{rcl}{\lambda }_{1} & 0 & 0\\ 0 & {\lambda }_{2} & 0\\ 0 & 0 & {\lambda }_{3}\end{array}\right){\left(\begin{array}{rcl}\vdots & \vdots & \vdots \\ {{\bf{v}}}_{1} & {{\bf{v}}}_{2} & {{\bf{v}}}_{3}\\ \vdots & \vdots & \vdots \end{array}\right)}^{-1}$$

Eigenvalues *λ*_*i*_, *λ*_*j*_ and *λ*_*k*_ were used to estimate the local fractional anisotropy (FA), defined as in equation (4):

4 $$\mathrm{FA}=\sqrt{\frac{1}{2}}\times \frac{\sqrt{{({\lambda }_{i}-{\lambda }_{j})}^{2}+{({\lambda }_{j}-{\lambda }_{k})}^{2}+{({\lambda }_{i}-{\lambda }_{k})}^{2}}}{\sqrt{{\lambda }_{i}^{2}+{\lambda }_{j}^{2}+{\lambda }_{k}^{2}}}$$

The FA provides a measure of the degree of anisotropy inside *p*. We set a threshold on the FA of 0.25 to include in the orientation map only *p* with sufficient anisotropy of cellular fluorescence signal. For each of them, we identified the eigenvector associated with the lowest eigenvalue, thus indicating the direction carrying the lowest gradient variability of the fluorescence signal. This was taken to be the principal direction of locally prevailing cardiomyocyte orientation (henceforth referred to as ‘fiber orientation’). The selected eigenvector was normalized and defined as the ’orientation vector‘. Iterating on the entire tomography, this vector space reassembles the fiber orientation architecture of the whole sample (Supplementary Fig. 13a). Cellular orientation maps were then inserted into geometrical models (tetmesh) by mapping each vector into the corresponding mesh element (Supplementary Fig. 13b). A rule-based cellular distribution was generated as well for both samples^69^. Supplementary Video 4 shows a geometrical model of a DSG2 heart, including the rule-based (on the left) and the image-based cellular orientation extracted from the fluorescence signal (on the right). The 3D cellular architecture was visualized in ParaView as colored glyphs or streamlines. The helix angle of each vector was calculated (and color-mapped) in ParaView. Supplementary Videos 5 and 6 show an image-based model of a DSG2 mouse heart: in Supplementary Video 5, the cellular helix angle is color coded and segmentations of CF and NCF are represented in black and gray, respectively; in Supplementary Video 6, cellular organization is represented by streamlines, and CF is shown in black.

### Cellular disarray quantification

We defined cellular disarray as the difference between the local fiber direction (100-µm scale) and the dominant fiber direction (300-µm scale). According to this, we computed 3D cellular disarray in a subset of samples (two CTRL, four DSG2). First, the dominant direction is obtained by applying a smoothing (that is, low-pass) filter to the fiber field. This filter serves to eliminate the rapid oscillations inside a virtual kernel of 300-µm width, which corresponds to the disarray intended to be quantified. The developed procedure combines several elements tailored to this operation, accounting for the peculiar characteristics of the data under consideration. Notably, it considers the directional invariance of the fiber field (for example, both the directions ***f*** and *-****f*** are identical), and its circular nature, where angles of 0° and 360° denote the same quantity in practice. The procedure encompassed the following steps:

- For each point of the tetmesh, an orthonormal local coordinate axial system, composed by the unit transmural **e**_t_, apico-basal **e**_n_ and circumferential **e**_l_ directions, was computed as described in Piersanti et al.^70,71^. Then, the local fiber orientation angle *α* was defined as the angle with respect to **e**_l_ in the plane (**e**_t_**e**_t_, **e**_n_) (see Piersanti et al.^70^ for more details).
- The angle *α* was mapped from $[0,\,2\uppi )$ to $[-\uppi /2,\,\uppi /2)$, thus defining a unique angle for each pair of equivalent directions, followed by the transformation ${{\eta }}=(\cos (2\alpha ),\,\sin (2\alpha ))$. This conversion from $[0,\,2\uppi )$ to the Cartesian plane *R*^2^ was consistent with both directional invariance and the circular topology of fiber orientation. Consequently, it transitioned to a more natural domain than $[0,\,2\uppi )$ for implementing smoothing techniques.
- A Laplacian smoothing was performed to yield a smoothed field, denoted as $\bar{{{\eta }}}$, computed as the solution of the equation $-{\delta }^{2}{{\Delta }}\bar{{{\eta }}}\,+\,\bar{{{\eta }}}\,=\,{{\eta }}$, equipped with homogeneous natural boundary conditions. The parameter *δ* represents the characteristic smoothing length (see Regazzoni et al.^72^ for further details). The solution is obtained through linear Finite Elements, by relying on the library life^x^ [https://lifex.gitlab.io/]^73^. Unlike smoothing methods defined in the frequency domain (which typically require the use of structured meshes), our approach can be seamlessly applied to data defined on arbitrary grids.
- Finally, the smoothed angle was retrieved by reversing the previously mentioned coordinate transformation: $\bar{\alpha }\,=\frac{1}{2}\mathrm{atan}2({{{\eta }}}_{{{1}}},\,{{{\eta }}}_{{{2}}})$. The angle $\bar{\alpha }$ represents the dominant fiber direction in each point of the domain. The local disarray is thus evaluated as the difference between the measured angle *α* and the dominant local fiber direction $\bar{\alpha }$, selecting the most coherent representation of α relative to $\bar{\alpha }$, determined by minimizing the difference among all equivalent representations of α, namely, $\mathop{\min }\limits_{k\in \{-\mathrm{1,0,1}\}}|(\alpha \,+k\uppi )\,-\,\bar{\alpha }|.$

### Morpho-functional co-registration

3D tomograms containing structural information were correlated with functional information from LV free wall optical mapping, by realigning virtual models to the original position of the Langendorff-perfused hearts during optical mapping. A Python pipeline was developed to this goal. First, a series of 3D rotations around the longitudinal (θ) and transverse (ϕ) axes (both ranging from –40° to +40°, in 10° increments) was automatically applied to the 3D segmentation of mesoSPIM fluorescence. For each (θ, ϕ) combination, average intensity projections were generated (Supplementary Fig. 5a,b) and compared with the fluorescence signal acquired during optical mapping (Supplementary Fig. 5c,d). In detail, both images (mesoSPIM average intensity projection and fluorescence from optical mapping) were segmented, and the heart long axis was realigned by estimating the principal direction of the image gradient. The pipeline automatically selected only the ventricular portion by cropping the upper 40% of the image (to avoid errors due to incomplete reconstruction of the heart base in mesoSPIM tomograms), and ventricles were shifted to align their centroids. Edges were extracted, and a set of rigid transformations (rotations from –30° to +30°, in 5° steps; translations from –20 to +20 pixels, in 5-pixel steps along both *x* and *y* axes) was applied to the mesoSPIM-derived edges. For each transformation, the average distance (Hausdorff distance) between edges was computed in micrometers. The minimum distance, indicating the best match possible between the two 2D shapes, was reported for each (θ, ϕ) rotation (Supplementary Fig. 5e), and the optimal (θ, ϕ) rotation (providing the minimum distance) was applied to the 3D mesoSPIM dataset. Supplementary Fig. 5f displays the scores in term of average distance of the automatic registration algorithm for each heart (the best fit is marked by a black circle), yielding an average difference across *N* = 9 DSG2 hearts of 85 μm ± 18 μm of ventricular shape between functional and structural imaging. Supplementary Fig. 6 shows the superimposition of fluorescence data from optical mapping with the 2D view of the mesoSPIM-based 3D dataset following registration (cardiac shape is preserved, and regions lacking VSD-related fluorescence correspond to scattering signals indicative of collagen presence).

For each heart, this registration pipeline was applied to the 3D model (biventricular mesh) representing cardiac anatomy in terms of myocardium, CF and NCF. Functional data derived from optical mapping (activation maps and delta maps) were then linearly projected onto the mesh surface (Fig. 5a and Supplementary Video 7). Finally, to generate morpho-functional correlation figures, only CF and NCF located within the optically mapped half-heart free wall were considered. For improved visualization of fibrosis distribution in Fig. 7a,c,e, CF and NCF are displayed as contour edges.

### Whole-ventricle electrophysiological simulations

Simulations were conducted on the image-based organ tetmesh using a monodomain model of electrophysiology in CARPentry-Pro (NumeriCor). Cellular ion flux dynamics were represented using the murine ventricular myocyte proposed by Li et al. (LiSmith model^74^). A 3D anisotropic conductivity tensor was used to replicate the fiber structure of the ventricles. To do so, tissue conductivity values along and transverse to the fiber direction were uniformly rescaled to approximate the total activation time observed in the optical mapping experiments during apical pacing at LF.

The CF was represented as being electrically nonconductive by imposing no-flux boundary conditions at its interface with surviving myocardium. NCF was modeled by assigning a subset of the mesh elements tagged as NCF to be non-conducting. Specifically, each NCF element was assigned as non-conducting with a probability proportional to the local scattering intensity at its spatial location. Regions exhibiting higher scattering signals—indicative of denser fibrotic tissue—were more likely to be modeled as non-conducting. This image-informed approach ensures that both the proportion and spatial distribution of non-conducting elements reflect the underlying tissue structure^22^.

To investigate the impact of functional alterations in the NCF region, we conducted additional simulations in which we modified local electrophysiological properties. We reduced tissue conductivity by setting both longitudinal and transverse conductivities to 25% of the control transverse value. We considered both isotropic and anisotropic configurations: in the latter, transverse conductivity was further reduced to 25% of the longitudinal value, maintaining the same anisotropy ratio used in our simulations of healthy myocardium (ratio of 4). Second, we impaired excitability in the NCF by decreasing the maximum sodium channel conductance to 25% of the control value. Third, we incorporated active fibroblasts into the NCF region by coupling each myocyte to 12 fibroblasts, modeled using the Morgan et al. formulation^75^. Finally, we evaluated a combined condition in which all three alterations—reduced conductivity (using the isotropic case), sodium current reduction and fibroblast coupling—were simultaneously applied.

Models were initialized with single-cell model states obtained after pacing the LiSmith murine ventricular cell model at LF (5 Hz) for 100 cycles. Electrical activity was initiated at the apex of all models to replicate the experimental pacing protocol. All models were paced for ten beats at LF, followed by another ten beats at HF (15 Hz), analyzing data during the 10th cycle for each mode of pacing.

To approximately replicate the averaged over tissue depth inherent to the optical mapping technique, data were averaged within a 1-mm layer below all epicardial surface nodes.

### Analysis of Cx43 distribution in two-photon reconstructions

We quantified the area characterized by punctate Cx43 using two-photon reconstructions of equatorial slices from cleared DSG2 (*N* = 2) and CTRL (*N* = 2) hearts. Analysis was performed in 2D at two different depths for each slice (300-μm thickness) using the Cx43 channel. For each plane, we randomly selected five ROIs of 887 µm × 887 µm from: fibrotic area of DSG2, remote area of DSG2 and healthy myocardium of CTRL. We identified a punctate Cx43 area through an optimized 2D segmentation procedure based on a neural network, involving the Trainable Weka Segmentation plugin available in Fiji. First, a subset of ROIs was randomly selected among all and used as a training set, where selected areas were manually classified to train the software in discriminating between Cx43 punctate area and linear morphology. The model assigns to each pixel of the ROI a probability *p* ∈ [0, 1] to be inside an area with punctate Cx43. Finally, the average probability was calculated for each ROI.

### 1D model of electrotonically coupled myocytes–fibroblasts

We modeled an electrotonically coupled 1D chain of ten myocytes, followed by *n* ∈ [1,9] fibroblasts, and a final downstream myocyte as a reporter of suprathreshold conduction via the non-excitable fibroblast insert. We used the Bondarenko ionic model^76^ for mouse cardiac myocytes, and the Sachse^77^ model for cardiac fibroblasts. For each run, we stimulated the initial five myocytes for 0.5 ms per stimulus with a current amplitude of 10 nA at different pacing rates (from 0.5 Hz to 19.5 Hz, incremented in 1-Hz intervals). For each pacing rate, we preconditioned the chains with 100 paced excitations to approach a limit cycle, as suggested in Sachse et al.^77^, and investigated the 20 subsequent cycles. For the last two preconditioning cycles, we confirmed cross-correlation of AP traces close to 1, which corroborated that the system had reached a limit cycle. For each combination of *n* and pacing rate, we assessed successful conduction by checking whether for each of the 20 cycles, an AP was triggered in the end-standing single cardiomyocyte. If one or more of the 20 excitation cycles was not captured by the downstream cardiomyocyte, we considered conduction to have failed. For each pacing rate, we determined the maximal *n* of insertable fibroblasts that still support sustained downstream excitation. For these combinations of pacing rate and *n*, we then assessed AP upstroke velocity of the end-standing myocyte ((d*V*/d*t*)_max_ in mV ms^−1^). Finally, for a fibroblast insert length of *n* = 4, we determined the pacing-rate dependency of conduction velocity between the proximal (last myocyte of the initial ten myocytes) and the end-standing myocyte. We normalized values by the conduction velocity at the slowest stimulus frequency (0.5 Hz).

### Image preprocessing and analysis

MesoSPIM datasets were originally acquired at a near-isotropic resolution of 3.25 × 3.25 × 3.1 µm³. For consistency across analyses, all volumes were first resampled to an isotropic voxel size of 6 × 6 × 6 µm³ using bilinear interpolation (ImageJ). This high-resolution dataset was used exclusively for the analysis of myocyte orientation, performed with a spatial resolution of 96 × 96 × 96 µm³. For segmentation and anatomical analysis, the data were further downsampled to an isotropic voxel size of 20 × 20 × 20 µm³ by a bilinear interpolation (ImageJ). This downsampling step was applied to reduce file size, suppress noise and minor artifacts, and improve processing efficiency. Anatomical meshes were generated by 3D segmentation, with an average element size of approximately 100 µm.

Image processing was performed with the Fiji distribution of ImageJ software (https://imagej.net/software/fiji/)^78^. Image segmentation was performed with Seg3D (CIBC: Seg3D: Volumetric image segmentation and visualization. Scientific Computing and Imaging Institute (SCI), 2016, https://github.com/SCIInstitute/Seg3D/releases). Unless stated otherwise, all other image-processing steps were performed automatically using software tools developed in Python 3.6.8. The main Python 3 libraries involved were: NumPy (1.17.3)^79^, SciPy (1.3.1)^80^ and Scikit-learn (0.16.2)^81^ for numeric elaboration, and Scikit-image (0.16.2)^82^, OpenCV-python (3.2.0.8)^83^, Tiffile (2019.7.26) and Pillow (6.2.1) for image processing.

### Statistics

For each experimental condition apart from the differential maps, each point represents data from one heart, which were used for comparison and statistical analysis. A two-way repeated-measures ANOVA test was used to compare electrophysiological features between CTRL and DSG2 mice at LF and HF. For the comparison of means, Tukey’s post hoc analysis was used. Arrhythmia inducibility in CTRL and DSG2 was compared using Fisher’s exact test. For the differential of APD_90_, TTP and local conduction time, two measurements per heart were performed and added to the dataset. The normality of data distribution was assessed by a Shapiro–Wilk test. According to normality, or lack thereof, an unpaired Student’s *t*-test was used for delta APD_90_ and TTP data, and a Mann–Whitney test was used on delta local conduction time data to assess differences between groups. A *P* value of <0.05 was considered indicative of a statistically significant difference between means. Statistical analysis was performed using GraphPad Prism, version 10.1.1.

### Reporting summary

Further information on research design is available in the Nature Portfolio Reporting Summary linked to this article.

## Supplementary information

Supplementary Information Supplementary Figs. 1–13.

Reporting Summary

Supplementary Table 1 Optimization of thresholding levels for collagen segmentation in CTRL hearts. For each CTRL heart (CH), the threshold value (TH) used to segment collagen from the scattering signal is reported, with the myocardium and the resulting collagen volumes. Threshold values were selected to fit an average percentage of segmented collagen in the organ of about 2–2.5%, as reported in the literature^66,67^.

Supplementary Video 1 Tomographic reconstruction of a cleared DSG2 mouse heart by means of mesoSPIM-based imaging and preprocessing steps (isotropic voxel size: 6 µm). Myocardial fluorescence and collagen-related scattering signals are shown in grey and red, respectively.

Supplementary Video 2 Three-dimensional rendering of a cleared DSG2 heart reconstruction. Myocardial fluorescence and collagen-related scattering signals are shown in grey and red, respectively. The tomogram is displayed longitudinally cropped (left) and transversally cropped (right).

Supplementary Video 3 Three-dimensional anatomical model (tetrahedral mesh; average element side length: 100 µm) of the ventricles of a DSG2 heart reconstructed using the mesoSPIM setup, including segmentation of myocardium (grey), compact fibrosis (black) and non-compact fibrosis (green).

Supplementary Video 4 Three-dimensional anatomical model of the ventricles of a DSG2 heart with embedded cardiomyocyte orientation. Two different approaches for define 3D cardiomyocytes orientation are compared: generated by a mathematical model (rule-based, left) and derived from the analysis of myocardial fluorescence signals (image-based, right). Myocardium is shown in transparent red, and cardiomyocyte orientation is represented by color-coded streamlines (helix angle from –80° to +60° relative to the heart’s longitudinal axis, blue to red).

Supplementary Video 5 Helix angle of image-based cardiomyocyte is color coded for each element of the anatomical model of a DSG2 mouse heart and shown in transverse slices together with the segmentation of compact fibrosis (black) and non-compact fibrosis (green dots). Myocardium is shown in transparent grey.

Supplementary Video 6 Image-based cardiomyocyte organization embedded into the anatomical model of a DSG2 mouse heart, represented by streamlines. The helix angle is color coded, and compact fibrosis is shown in black. A zoomed-in view highlights the epicardial region surrounding the compact fibrosis present in the left ventricular free wall.

Supplementary Video 7 Projection of optical mapping data onto the 3D anatomical model for morpho-functional correlation. An example of a 2D map of ventricular activation after apical pacing is overlaid on the 3D reconstruction of the same heart (myocardium in grey) using the proposed structure–function registration algorithm.

Supplementary Video 8 Optical mapping of a DSG2 heart showing conduction failure and re-entrant arrhythmia induced by premature beats. The heart was electrically stimulated at the right ventricular outflow tract during sinus rhythm. Resting and depolarized membrane potentials are displayed in green and red, respectively (frame rate: 1 ms). Heart shape and compact fibrosis are outlined in grey and white, respectively. The video first shows a sinus beat followed by a delayed electrical stimulus propagating through the fibrotic area. A second stimulus delivered immediately after a sinus beat generates a conduction wavefront distorted by fibrotic tissue, ultimately triggering re-entrant arrhythmia.

Supplementary Video 9 Optical mapping of a DSG2 heart showing conduction failure and re-entrant arrhythmia induced by premature beats in a different DSG2 heart with respect to Supplementary Video 8. The heart was electrically stimulated at the right ventricular outflow tract during sinus rhythm. Resting and depolarized membrane potentials are displayed in green and red, respectively (frame rate: 1 ms). Heart shape and compact fibrosis are outlined in grey and white, respectively. The video shows an electrical stimulus delivered immediately after a sinus beat that generates a conduction wavefront distorted by fibrotic tissue, triggering re-entrant arrhythmia.

## Source data

Source Data Fig. 1 Statistical source data.

Source Data Fig. 3 Statistical source data for Fig. 3b.

Source Data Fig. 3 Statistical source data for Fig. 3e.

Source Data Fig. 5 Statistical source data.

Source Data Fig. 7 Statistical source data.

Source Data Fig. 8 Statistical source data for Fig. 8h.

Source Data Fig. 8 Statistical source data for Fig. 8g.

Source Data Extended Data Fig./Table 7 Statistical source data.

## Data Availability

Functional and structural datasets generated and analyzed during the current study are available at 10.60493/9zgpg-mb093 (ref. ^84^).

## Code availability

The Python source codes developed to analyze data during the current study are available at https://github.com/francescogiardini/heart.git/.
