# Supplementary material for: Correlative imaging integrates electrophysiology with three-dimensional murine heart reconstruction to reveal electrical coupling between cell types
Source: Nat Cardiovasc Res. 2025 Oct 6;4(11):1466–86. doi: 10.1038/s44161-025-00728-9 (PMC12611784; doi:10.1038/s44161-025-00728-9)
Supplement: Supplementary file 1 — Supplementary Figs. 1–13. [file 44161_2025_728_MOESM1_ESM.pdf]

nature cardiovascular research

Supplementary information https://doi.org/10.1038/s44161-025-00728-9

Correlative imaging integrates electrophysiology with three-dimensional murine heart reconstruction to reveal electrical coupling between cell types

In the format provided by the authors and unedited

CTRL

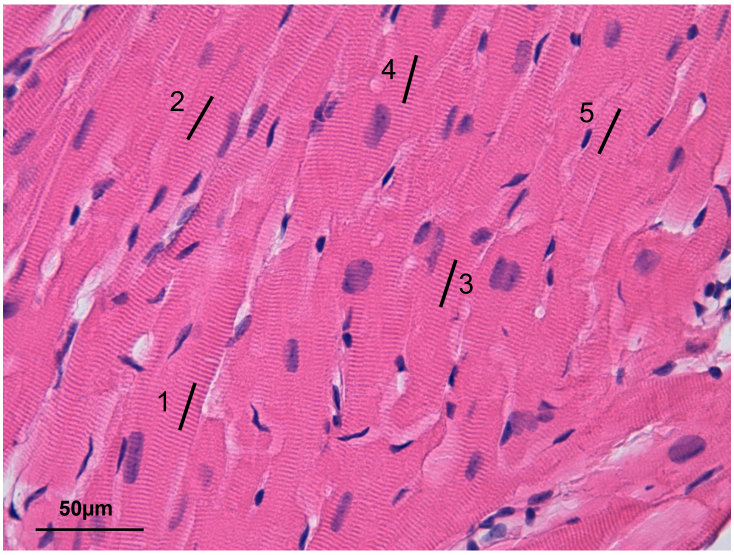

Sarcomere lengths:
1: 2.266 µm
2: 2.238 µm
3: 2.236 µm
4: 2.230 µm
5: 2.248 µm

Average: 2.24 µm
Std.dev: 0.01 µm

DSG2

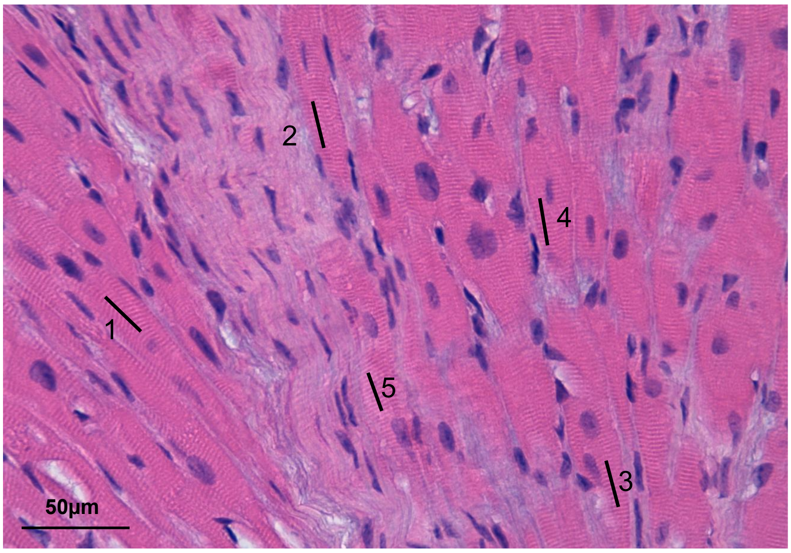

Sarcomere lengths:
1: 2.334 µm
2: 2.098 µm
3: 2.194 µm
4: 2.159 µm
5: 2.008 µm

Average: 2.16 µm
Std.dev: 0.11 µm

Supplementary Figure 1: Tissue preservation of clearing by SHIELD protocol. Results of the histological inspection of post-clearing murine myocardium tissue from a control and a DSG2 heart, performed using haematoxylin-eosin staining. Average sarcomere length (black lines, ten consecutive sarcomere z-lines) was measured by selecting cells with their major axis as parallel as possible to the image plane, in order to minimize estimation errors of sarcomere length (SL) caused by cell tilt in 3D space (cosine-error).

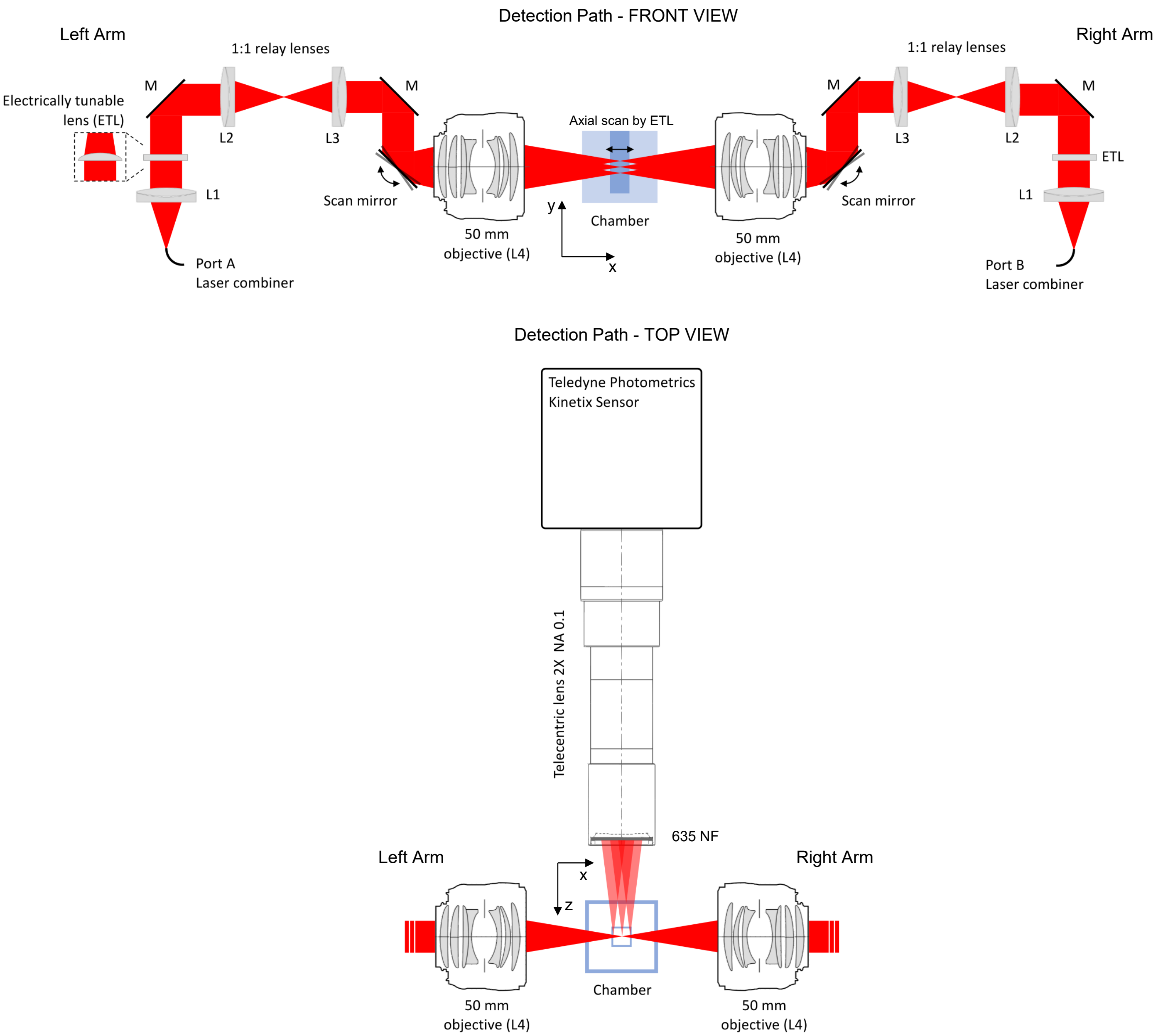

Supplementary Figure 2: MesoSPIM optical scheme. L1: Achromatic doublets (AC254-050-A-ML, Thorlabs), ETL: Electrically Tuneable Lens (EL-16-40-TC-VIS-5D-1-C, Optotune), M: Dielectric Mirror (BBE1-E02, Thorlabs), L2 and L3: Achromatic doublets (G063200000, Qioptiq), Scanning mirror (GVS211/M, Thorlabs), L5: Camera objective (Nikkor AF-S 50mm f/1.4G, Nikon), 650 NF: NF03-405/488/561/635E-25, StopLine quad-notch filter, Semrock.

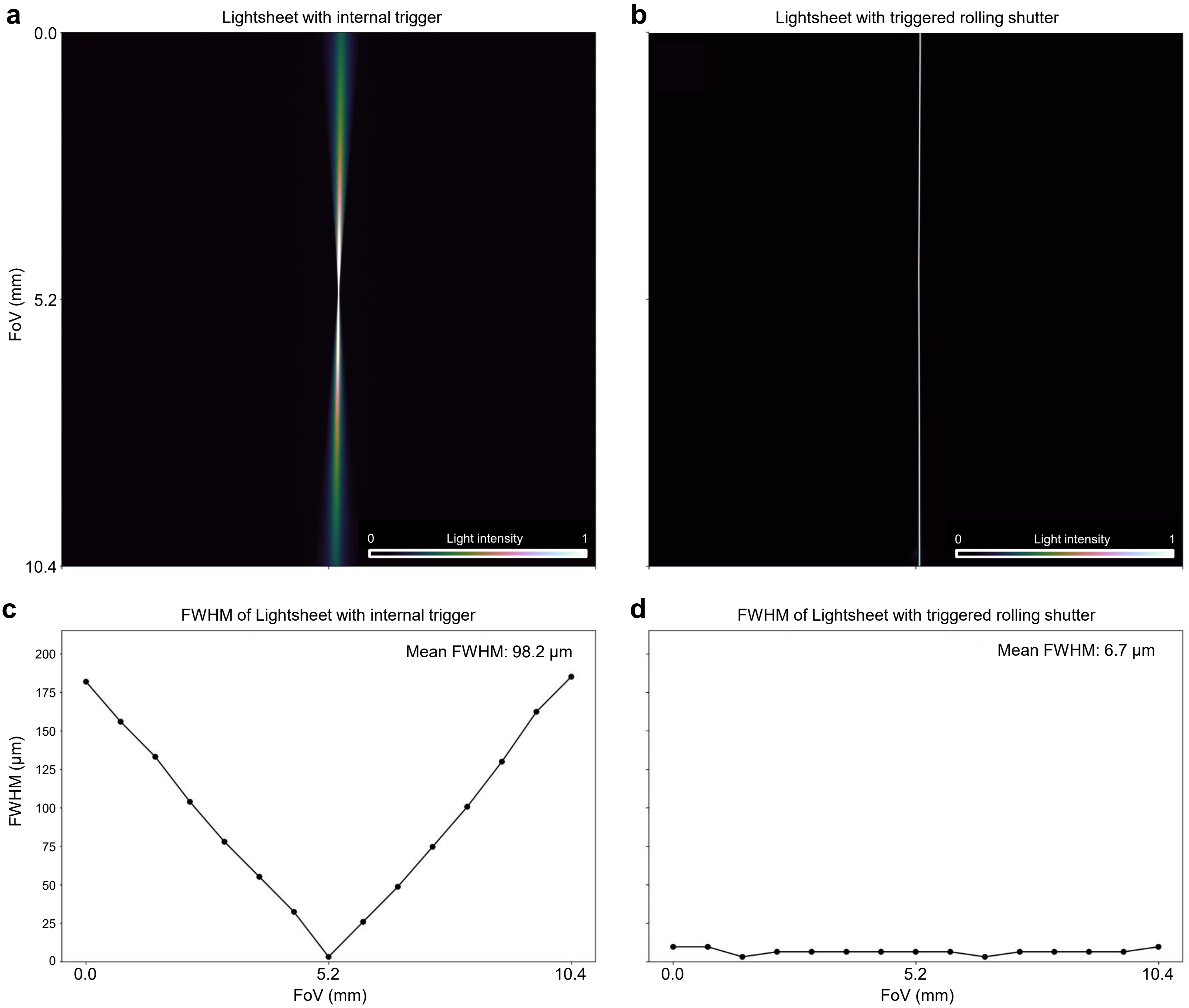

Supplementary Figure 3: MesoSPIM light-sheet generation. (A, B) Excitation light-sheet statically focused into the centre of Field of View (A) and dynamically generated by the synchronization between the camera rolling shutter (operating at 1.92 Hz) and the scanning light-beam driven by the tuneable lens (B). Pixel size: 3.25 µm; exposure time: 10 ms; light source: 638 nm; light intensity is normalized and reported with a colormap. The Full Width at Half Maximum (FWHM) of the light intensity profile is evaluated in 15 different positions along the FoV of panel A and B; results and averages are shown in panel C and D, respectively.

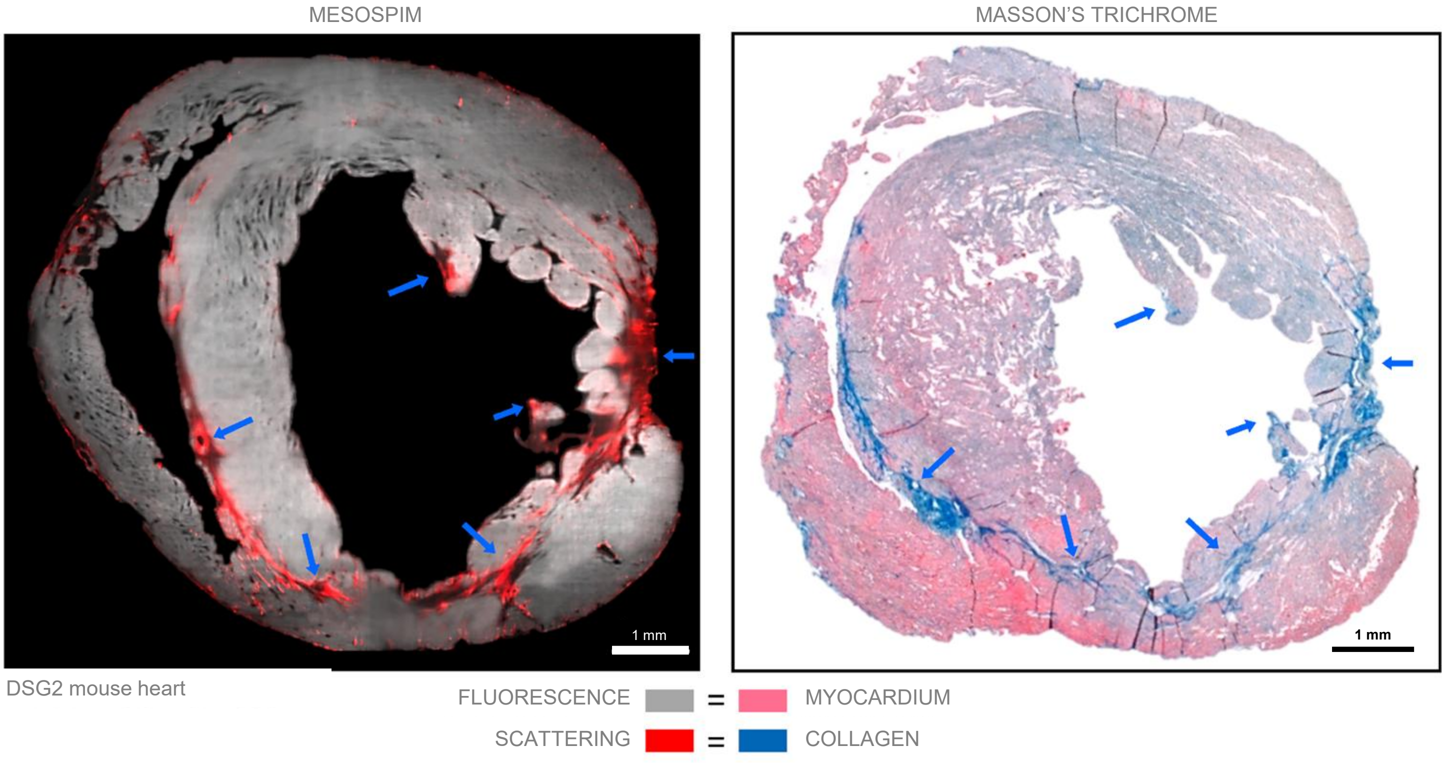

Supplementary Figure 4: Validation of the scattering signal by histology. On the left, a virtual section of a mesoSPIM-based tomography of a DSG2 mouse heart, where fluorescence signal (in grey) and scattering signal (in red) are shown. On the right, a Masson's trichrome staining of the correspondent histological section of the same heart highlights myocardial tissue (in pink) and collagen deposition (in blue). Histology confirms the accuracy of the proposed scattering-based approach to detect fibrotic areas (blue arrows).

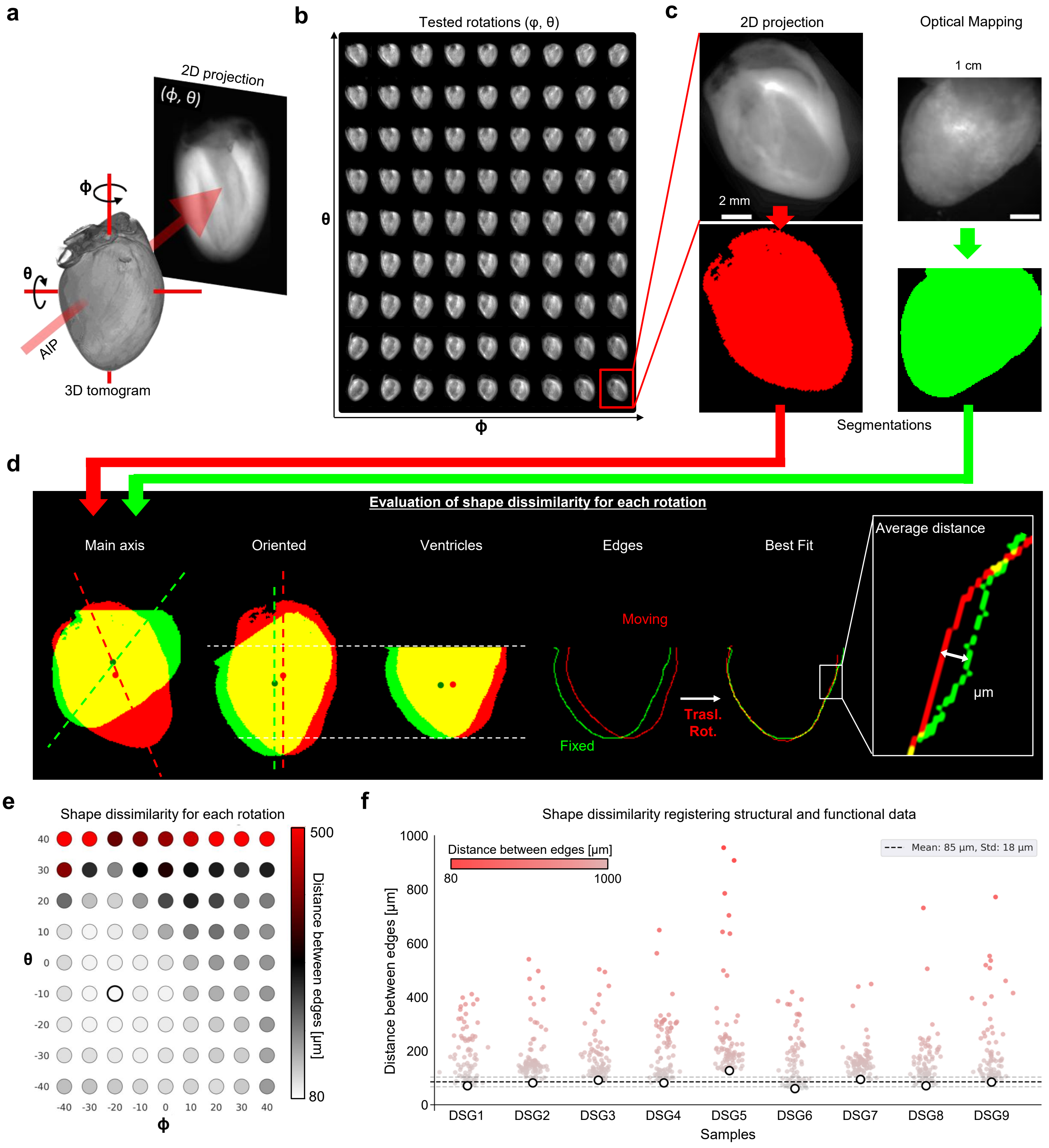

Supplementary Figure 5: Automatic registration pipeline between functional (2D) and mesoSPIM (3D) data based on heart shape. A: Schematic of the software tool used to generate Average Intensity Projections (AIP) of mesoSPIM-based myocardium tomographies, rotated around the long (φ) and transverse (θ) axes. B: Example of AIP for each (φ, θ) pair applied to a DSG2 heart tomogram (DSG9 sample), both ranging from –40° to +40°, with 10° increments. C: For each rotation, the AIP is segmented and compared to the segmentation of fluorescence signals acquired during optical mapping of the same heart. D: scheme of the automatic workflow estimating shape dissimilarity between mesoSPIM and OM heart views. The main axis of both segmentations is aligned, ventricular borders are extracted, and the mesoSPIM-based contour is rigidly transformed (rotation + translation) to minimize the mean distance between edges (best fit), calculated in micrometres (average distance). E: Results of the registration for a DSG2 heart, showing the minimum average edge distance for each tested (φ, θ) rotation. The configuration with the minimal distance (black-edged dot) is selected as the optimal rotation to apply to 3D structural data for morpho-functional correlation. F: Results across all DSG2 hearts (N=9). Each point represents the average distance for each tested rotation (φ, θ). White dots indicate the distance obtained with the optimal rotation. The dashed horizontal lines show the mean (black) ± standard deviation (grey) of the best scores across samples (N=9; 85 µm ± 18 µm).

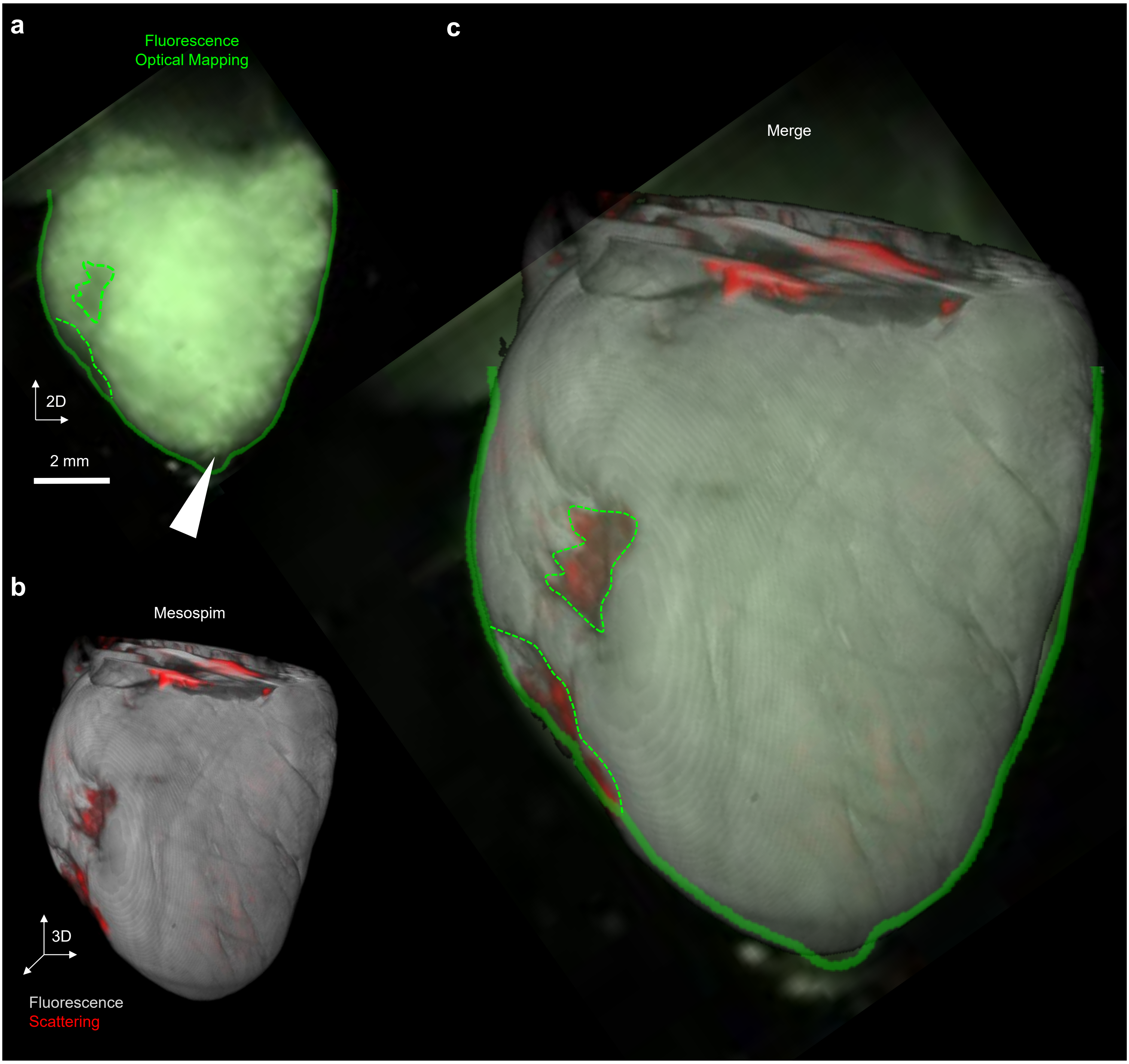

Supplementary Figure 6: Anatomical registration between functional and structural data. A: Fluorescence signal collected during optical mapping (shown in green; the ventricular shape is highlighted by edges, and dashed lines indicate a decrease in fluorescence signal, suggesting a lack of cells) of a DSG2 mutant mouse heart. The white arrow indicates the position of the electrode during apex pacing. B: 3D rendering of the same heart obtained by combining tissue clearing and mesoSPIM imaging, registered to the optical mapping data using the automatic software tool developed in this study (Fig. S13).

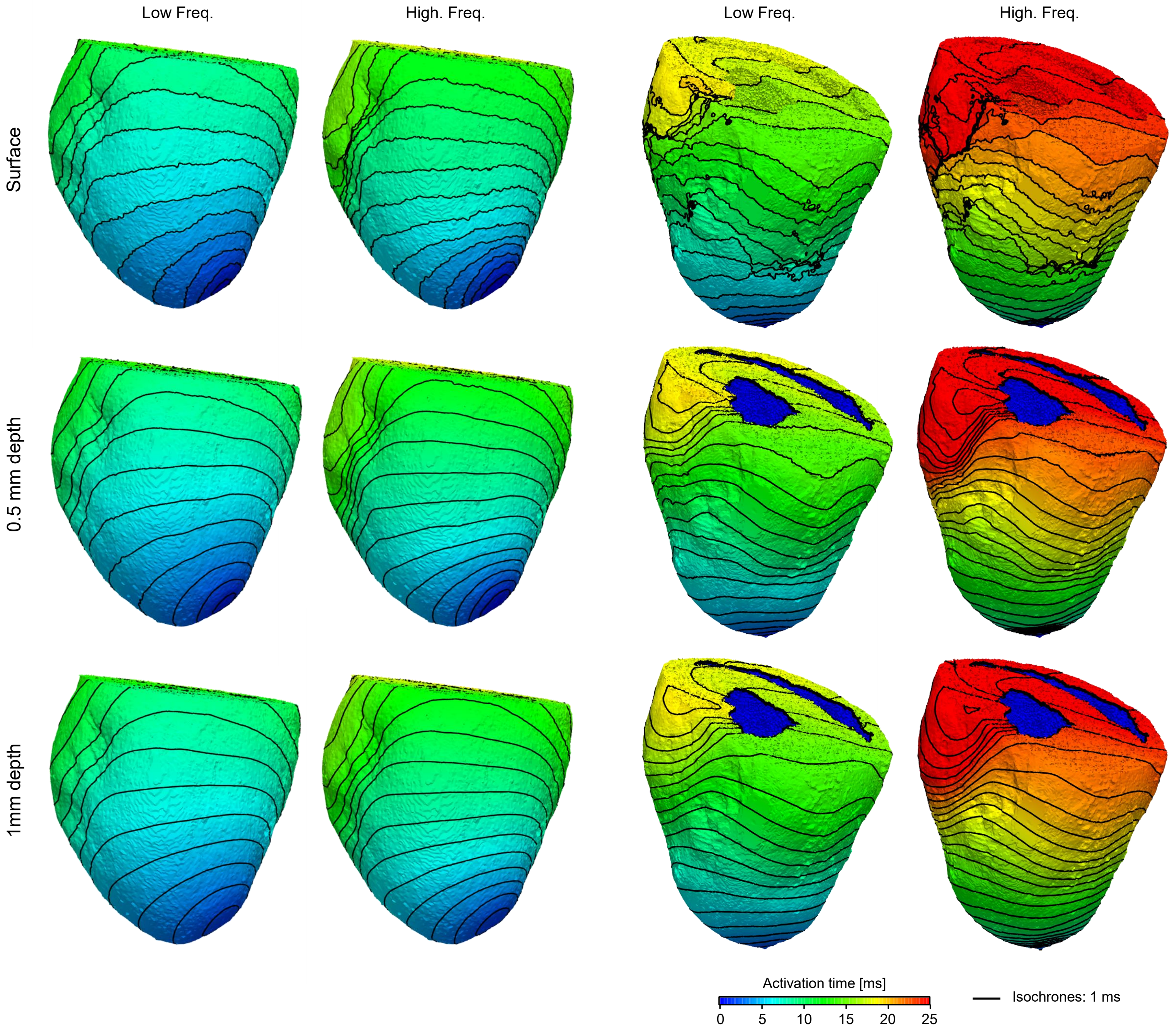

Supplementary Figure 7: Reconstruction of optical mapping signals from 3D simulations. Action potential propagation following apical pacing at low and high frequency is shown for one CTRL and one DSG2 heart in three different modality: activation time of only the outermost mesh elements; averaging over a depth of 0.5 mm; and averaging over 1 mm (full wall thickness).

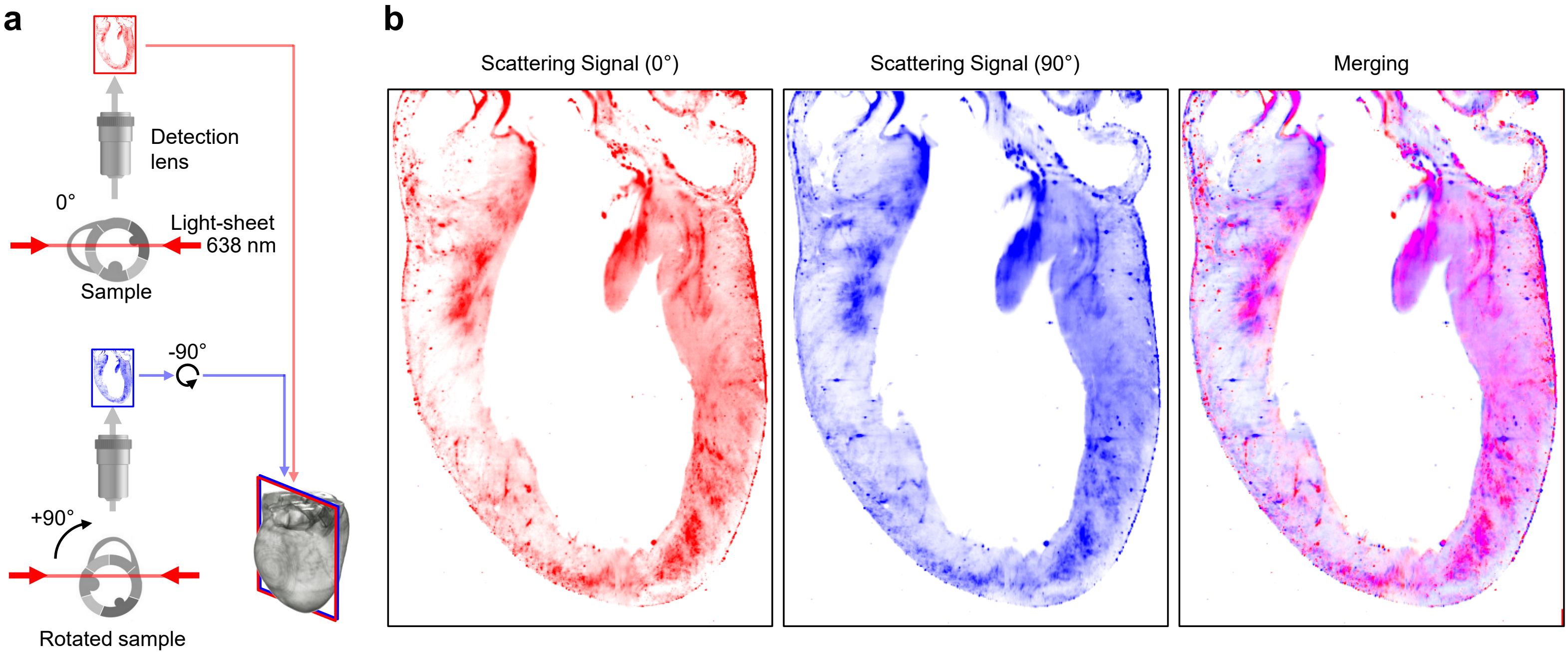

Supplementary Figure 8: Scattering signal independency from excitation direction. (A) Schematic diagram of the excitation and detection paths in two perpendicular configurations. (B) Representative tomographic internal sections of the scattering signal of the same heart, collected at 0° and 90° and subsequently realigned, both separated and merged.

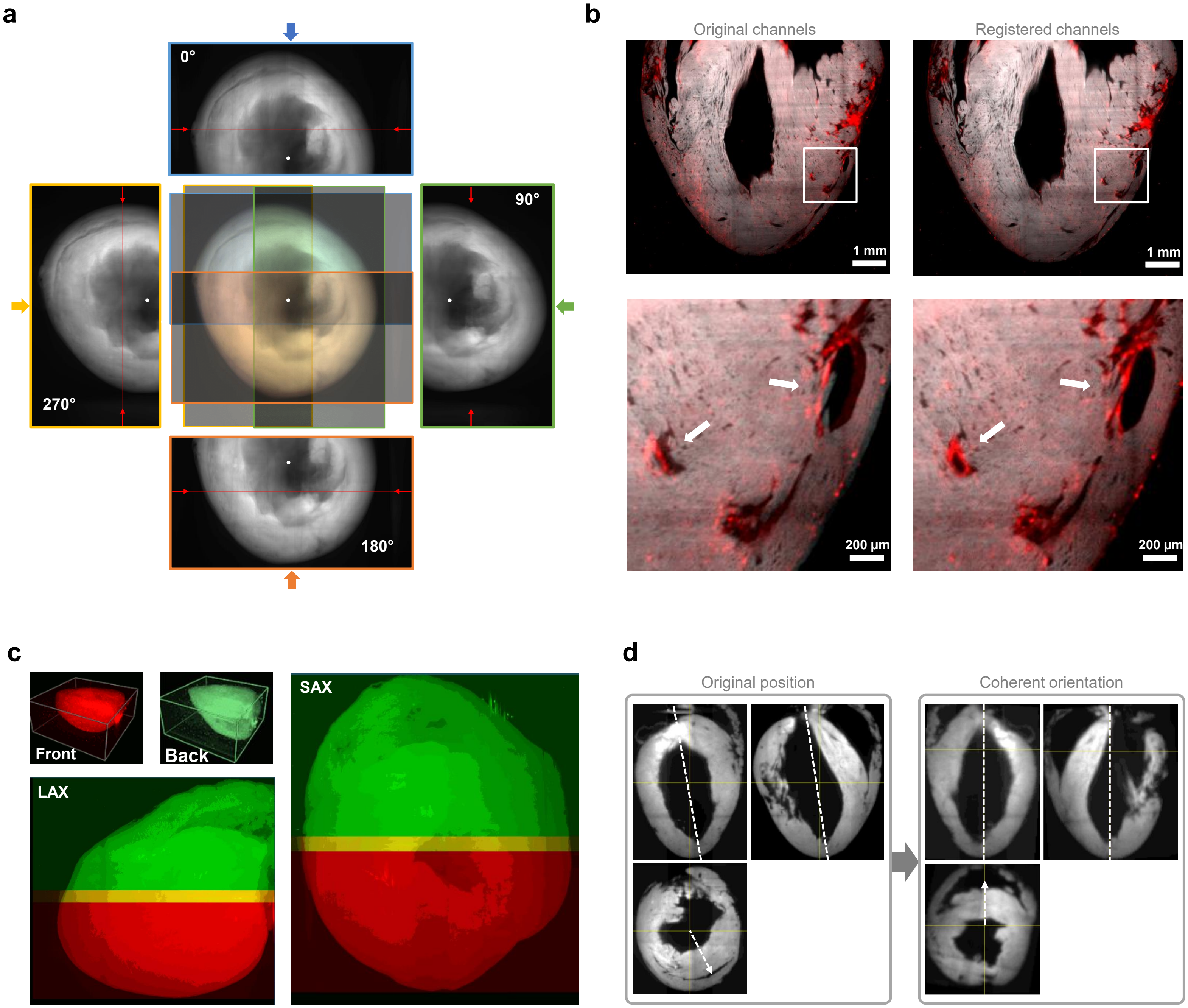

Supplementary Figure 9: Sample imaging and image preprocessing. (A) Schematic of multiple acquisitions performed to reconstruct an entire cleared mouse heart with the mesoSPIM setup. Partial tomographies were performed for each rotation of the organ around its long axis (white spots superimposed on Short-Axis Average-Intensity-Projections) at 0°, 90°, 180°, and 270° degree (blue, green, orange and yellow arrows indicate the optical axis). Red lines represent the optical sectioning plane of the light-sheet generated by a light source set at 638 nm from two parallel excitation arms (red arrows). In the center, the sample shape is reassembled by merging the four views as an example. (B) Result of the 3D channel registration process performed on a representative mouse heart reconstruction (muscle fluorescence in grey, scattering light in red) with the Fijiyama plugin (ImageJ) to correct the spatial shift (white arrows) between channels. (C) Registration of two opposite partial tomographies (front and back) of a reconstruction using Huygens Professional (SVI). After the initial manual registration, the software optimizes the alignment and fuse the 3D images. (D) Representative example of manual three-dimensional realignment performed on all reconstructions. The sample is rotated iteratively on each view of the 3d tomography to re-align the long axis of the organ and place the right ventricle on top of the SAX view.

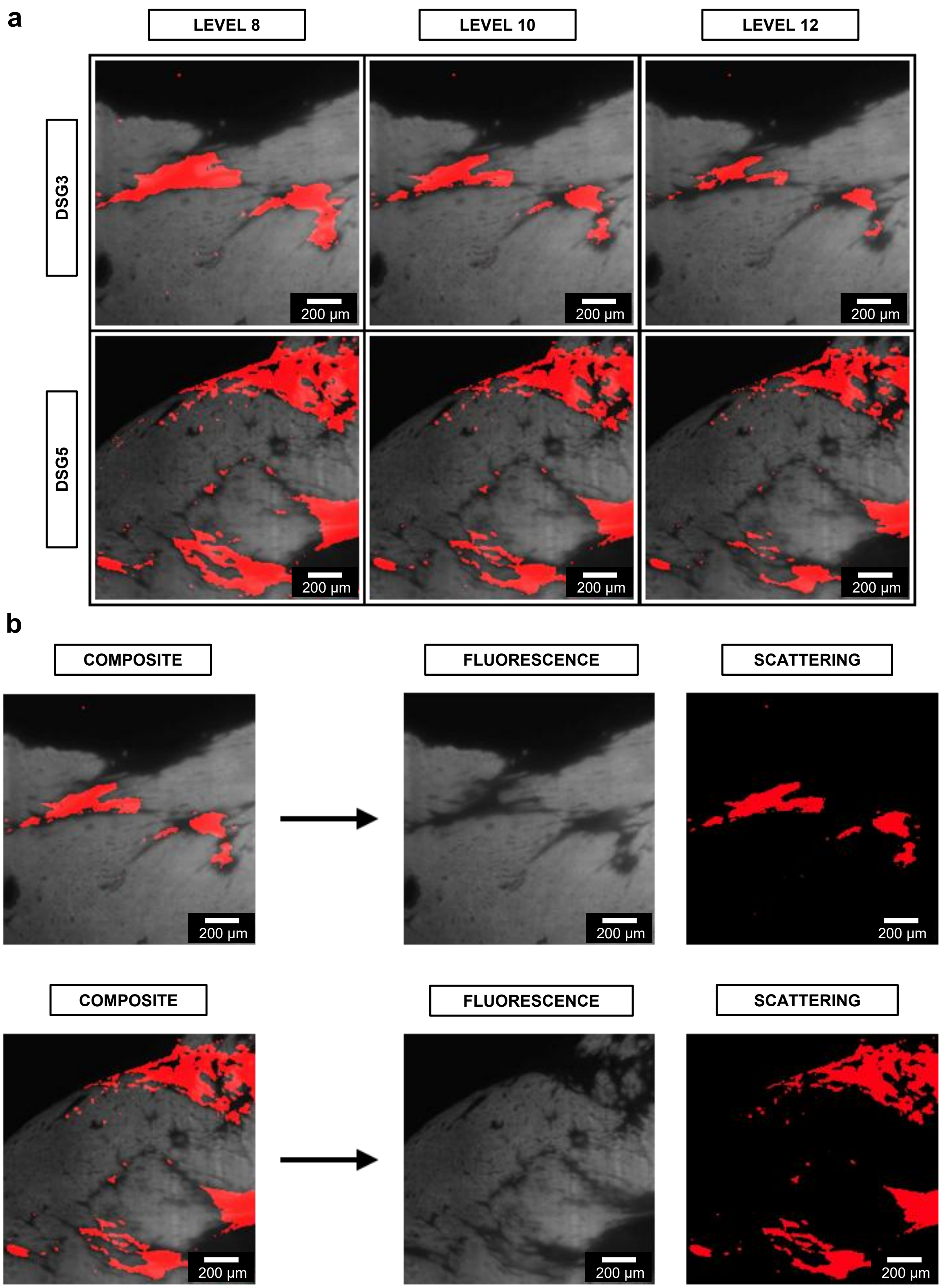

Supplementary Figure 10: Optimization of threshold-based segmentation of compact fibrosis from scattering signal. (A) Segmentation of compact fibrosis (CF) from scattering signal in regions of interests (ROIs) of two different DSG2 hearts (N3 and N6), varying the threshold of pixel intensity (level of gray, LEVEL). (B) Same ROIs shown in panel A, for intensity level = 10, showing the fluorescence and the scattering channels separately.

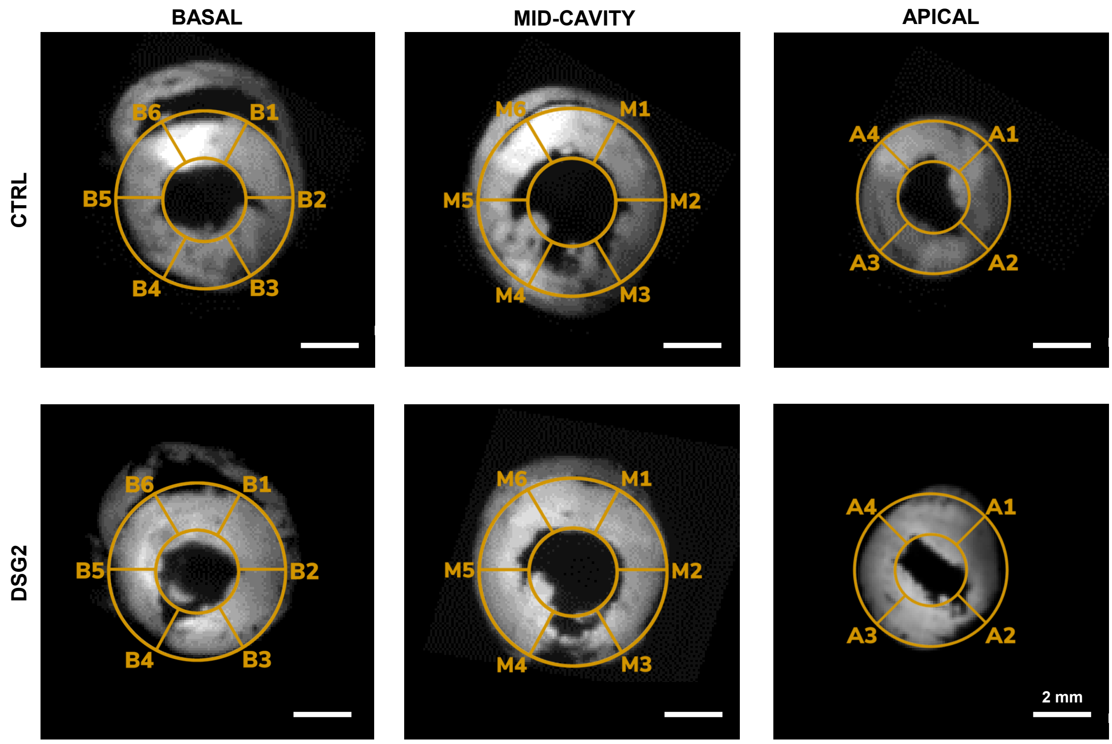

Supplementary Figure 11: Reference system for anatomical measurements of the Left Ventricle wall thickness. Representative Short-Axis views of fluorescence signal (in grey) of CTRL and DSG2 heart reconstructions used to measure LV wall thickness following the AHA seventeen-segments standard protocol (superimposed in yellow).

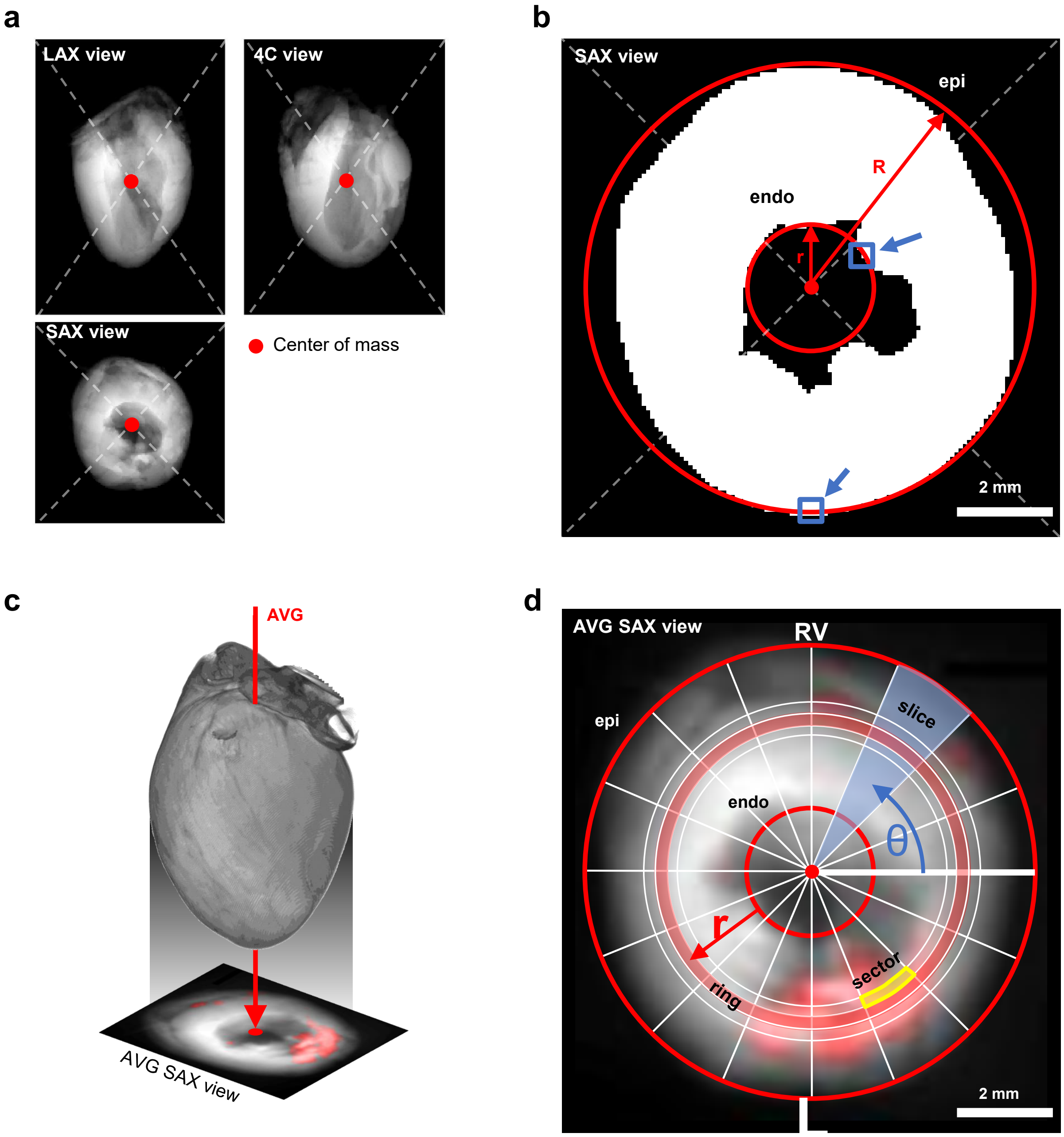

Supplementary Figure 12: Automatic spatial quantization of 3d data around LV wall. Representative schematics of the main steps of automatic quantization software developed to analyze the spatial distribution of fibrosis around LV wall in 3D segmentations. (A) The myocardium is centered in the 3d volume by evaluating the barycenter of the fluorescence signal. (B) The radii of circles that fit endocardium (r) and epicardium (R) surfaces are estimated matching smaller and bigger circumferences touching a pixel (blue arrows) of the myocardium segmentation in the central SAX view. (C) Segmentations were averaged along the main axis of the hearts, generating an average SAX (AVG SAX) view. (D) Angular and radial quantizations of the AVG SAX view are performed with sixteen incremental angles and sixteen incremental radii, respectively. The sum of the signal inside each sector is stored in a normalized LV map. Abbreviations: LAX: long-axis view, SAX: short-axis view, 4C: four-chamber, epi: epicardium, endo: endocardium, LV: left ventricle, RV: right ventricle, AVG: average.

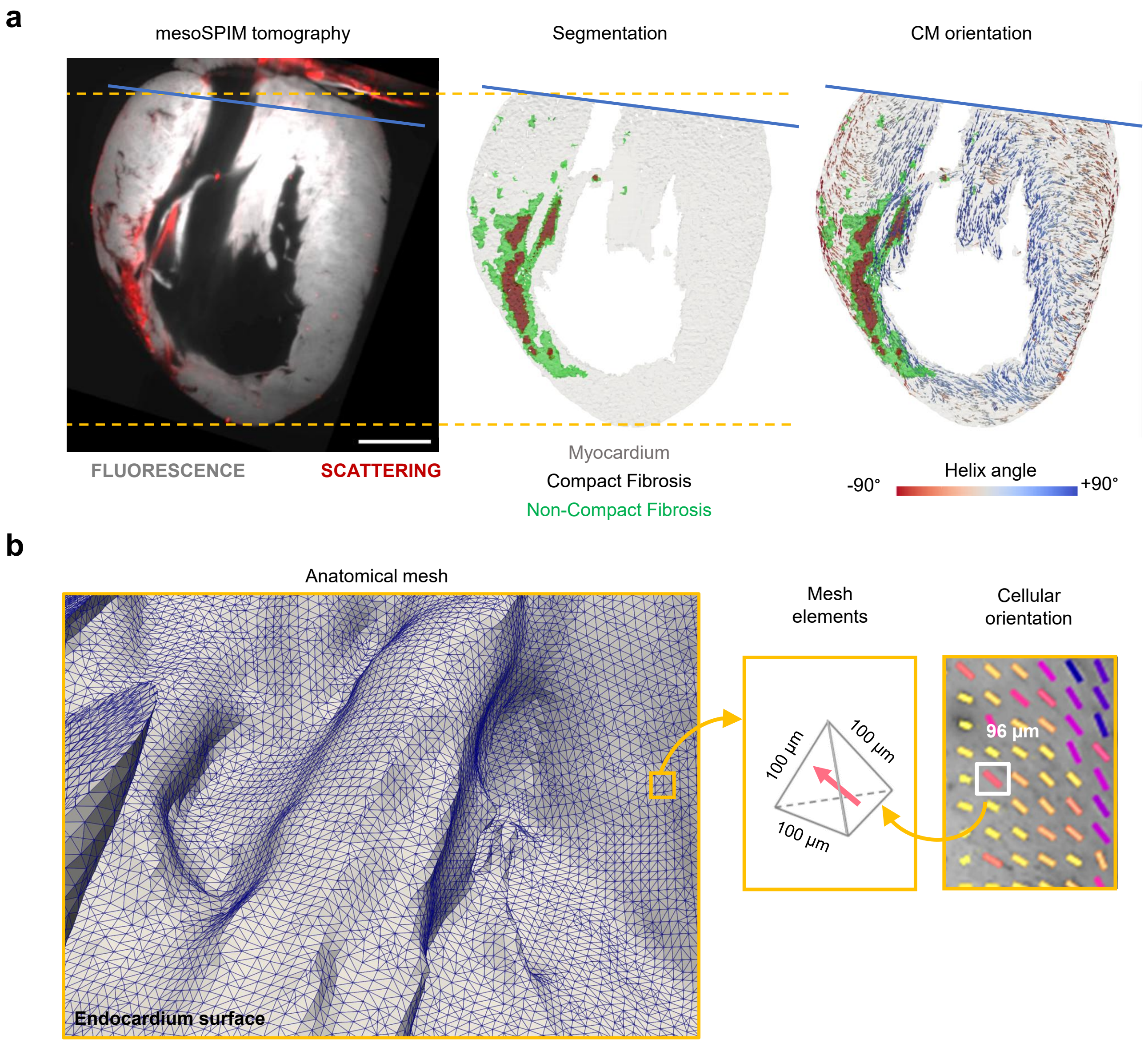

Supplementary Figure 13: Integrated anatomical model. (A) Workflow of integrated anatomical model generation. (B) Zoom of the endocardial surface of an anatomical model of a mouse heart, generated by the segmentation of mesoSPIM images. A single mesh element is represented by a tetrahedron, within which the local orientation of cardiomyocytes, extracted from the analysis of the myocardial fluorescence signal, is integrated.
